# Scalable, data-assimilated models predict large-scale shoreline response to waves and sea-level rise

**DOI:** 10.1038/s41598-024-77030-4

**Published:** 2024-11-14

**Authors:** Sean Vitousek, Kilian Vos, Kristen D. Splinter, Kai Parker, Andrea O’Neill, Amy C. Foxgrover, Maya K. Hayden, Jennifer A. Thomas, Li Erikson, Patrick L. Barnard

**Affiliations:** 1 U.S. Geological Survey, Santa Cruz, USA; 2 University of Illinois at Chicago, Chicago, USA; 3 UNSW Sydney, Sydney, Australia

**Keywords:** Climate-change impacts, Ocean sciences, Natural hazards

## Abstract

Coastal change is a complex combination of multi-scale processes (e.g., wave-driven cross-shore and longshore transport; dune, bluff, and cliff erosion; overwash; fluvial and inlet sediment supply; and sea-level-driven recession). Historical sea-level-driven coastal recession on open ocean coasts is often outpaced by wave-driven change. However, future sea-level-driven coastal recession is expected to increase significantly in tandem with accelerating rates of global sea-level rise. Few models of coastal sediment transport can resolve the multitude of coastal-change processes at a given beach, and fewer still are computationally efficient enough to achieve large-scale, long-term simulations, while accounting for historical behavior and uncertainties in future climate. Here, we show that a scalable, data-assimilated shoreline-change model can achieve realistic simulations of long-term coastal change and uncertainty across large coastal regions. As part of the modeling case study of the U.S. South Atlantic Coast (Miami, Florida to Delaware Bay) presented here, we apply historical, satellite-derived observations of shoreline position combined with daily hindcasted and projected wave and sea-level conditions to estimate long-term coastal change by 2100. We find that 63 to 94% of the shorelines on the U.S. South Atlantic Coast are projected to retreat past the present-day extent of sandy beach under 1.0 to 2.0 m of sea-level rise, respectively, without large-scale interventions.

## Introduction

Coastal evolution is a complex combination of many fine-scale hydrodynamic and geomorphic processes, each with characteristic spatiotemporal scales^1,2^. Models of coastal evolution, however, cannot capture all processes and scales^3,4^. Hence, many different modeling paradigms (e.g., 1-D vs. 2-D vs. 3-D and data-driven vs. reduced complexity vs. fully physics-based vs. hybrid models) exist to simulate site- and scale-specific behavior^4–12^.

Two- and three-dimensional (2-D and 3-D, respectively) physics-based, coupled coastal/ocean and sediment-transport models (also called ‘microscale’ models by Wolinsky^13^) are well-suited to simulating coastal change due to storm events, however, they are (and will likely remain) ill-suited to simulate large-scale (> 1000 km), long-term (10–100 year) coastal evolution due to their computational burden. We show that medium-resolution 2-D and 3-D physics-based models are approximately five orders of magnitude (i.e., 10,000x) more computationally expensive than a reduced-complexity model for simulations of large-scale (> 1000 km), long-term (10–100 year) behavior (see Supplementary Materials S1–S3 and its supporting analysis). Assuming that a large-scale, long-term physics-based simulation could be achieved from a computational standpoint with 2-D and 3-D models, it still remains unclear if the physics-based simulation would provide improved accuracy over a reduced complexity model when comparing models to observations^3^. Thus, we argue that scalable, data-integrated, reduced-complexity models or hybrid models^12,14,15^ are better suited (or are perhaps even mandatory, due to contemporary computational constraints) to reliably predict coastal change at real-world sites across large regions. In support of this argument, we present a modeling study of beaches along the U.S. South Atlantic Coast (Miami, Florida to Delaware Bay) with scalable, data-integrated, reduced-complexity model compared to historical shoreline observations across the region derived from satellites (see Results).

Reduced-complexity models^3^ are becoming ubiquitous in Earth sciences, and for good reason: computation constraints mean that models generally cannot resolve all relevant physics and/or scales for a given problem, and thus difficult trade-offs must be made. When modeling coastal change, one of the most important trade-offs is to *resolve vs. not to resolve* hydrodynamic processes and scales^4^. Hydrodynamic processes (e.g., waves, currents, and water levels) are strong drivers of coastal sediment transport, and, hence, they are often important to resolve^9^, especially when modeling individual storm events. Yet, the characteristic spatiotemporal scales of hydrodynamic processes (i.e., centimeters to meters and seconds to hours) are typically much shorter than scales associated with coastal morphologic change (i.e., meters to kilometers and days to years)^4^. Reduced-complexity/hybrid models often circumvent the need to resolve hydrodynamic processes and scales, via approximations (e.g., dimensional/physical reductions, idealizations, parameterizations, statistical transformations^16^ that seek to directly account for morphologic processes, driven by a set of forcing conditions, e.g., parameterized wave conditions). Furthermore, reduced-complexity models are well-suited to run in an ensemble^10,17,18^, which helps to understand the sensitivity of model inputs (e.g., physical drivers, parameter values, or anthropogenic factors) to outputs (e.g., model state variables, uncertainty estimates, vulnerability/risk/etc.). One of the greatest advantages of reduced-complexity/hybrid models, as exemplified here, is their ability to leverage time series of observations of critical processes and features (e.g., shoreline position) to calibrate and validate models^10,12,19–21^. The modeling study of the U.S. South Atlantic Coast presented below, which was part of a larger long-term coastal-hazard modeling effort for the U.S.^22–24^, demonstrates that accurate, quantitative predictions of coastal change can be achieved across a large scale with a reduced-complexity model (CoSMoS-COAST—Vitousek et al.^12,18,21^). Nevertheless, state-of-the-art reduced-complexity/hybrid models can underperform in certain complex coastal environments (e.g., small barrier islands) and for certain time scales, which may be better suited to fine-scale 2-D and 3-D physics-based models of limited spatiotemporal extent.

## Results

### Modeling shoreline change on the U.S. South Atlantic Coast using CoSMoS-COAST

**Fig. 1:**
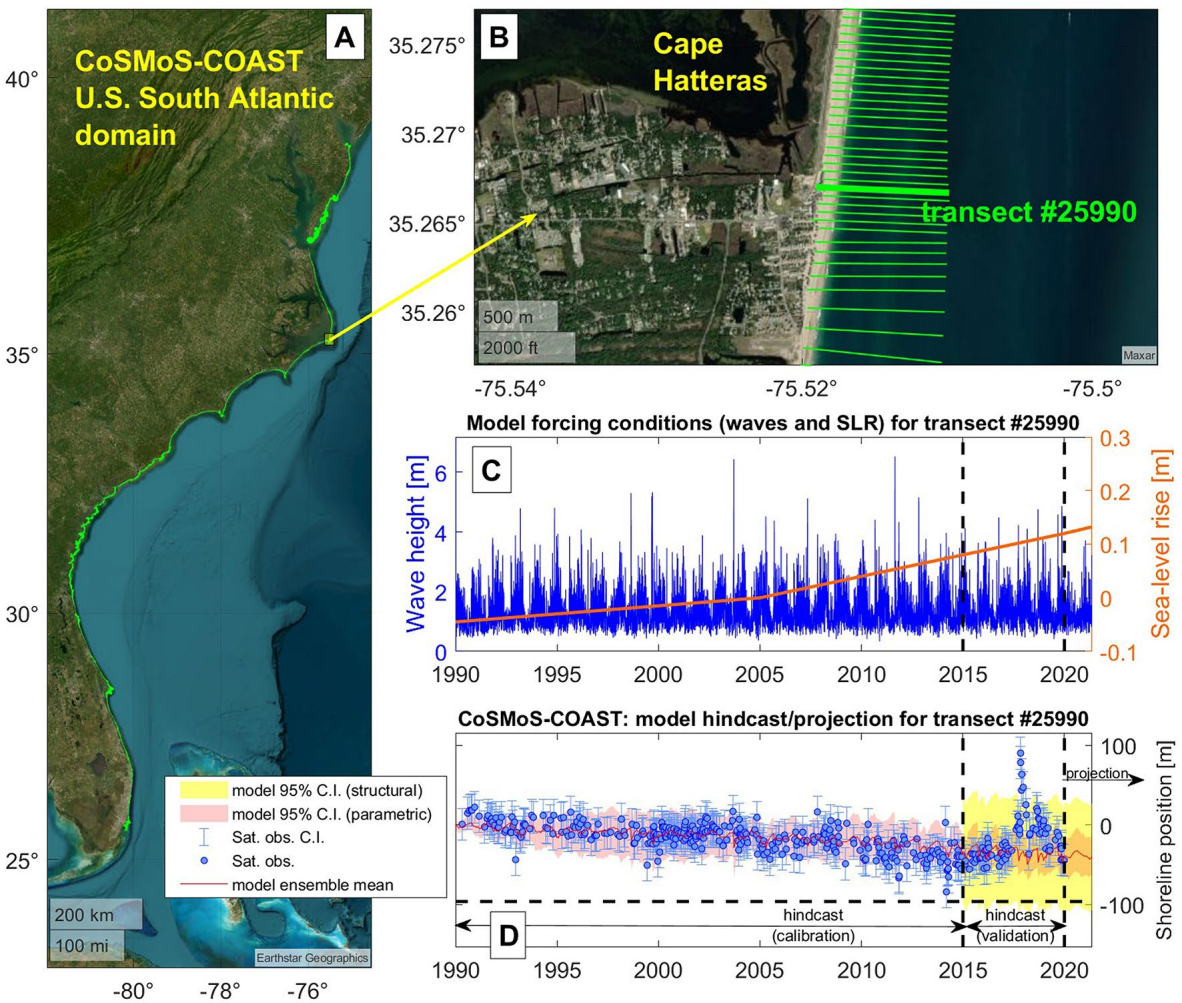
An example of calibration and validation of CoSMoS-COAST using historical satellite-derived shoreline data. The figure shows the extent of the CoSMoS-COAST U.S. South Atlantic Coast model transects (panel A—in green) with a zoomed in section of Cape Hatteras, North Carolina (panel B), which shows a close-up of the 50 m transect spacing (green lines). Panel C shows hindcasted and projected wave (blue line) and sea-level rise conditions (orange line) applied to the model at transect #25990 (i.e., the thick green line in panel B). Panel D shows the modeled shoreline position (red line) and its parametric uncertainty (pink bands), which are calibrated (from 1990–2015) with satellite-derived shoreline observations (blue circles) and their confidence intervals (blue ‘whiskers’). Later in the simulation, during the model “hindcast (validation)” period (2015–2020), data assimilation is turned off, and the calibrated model is compared to satellite-derived shoreline observations (blue circles) to assess the model’s structural uncertainty ($\pm 2\varepsilon_{{{\text{RMSE}}}}$, where $\varepsilon_{{{\text{RMSE}}}}$ is the model root-mean square error (RMSE) calculated using Eq. (2) and is shown in yellow confidence bands), which is propagated forward with a constant width during the model ‘projection period’ (2020–2100). Note that anomalies in shoreline position during the “hindcast (validation)” period, e.g., the accretion spike in 2017–2018 in panel D (caused by a beach nourishment), generally increase the model’s structural uncertainty (yellow bands) relative to the parametric uncertainty (pink bands). (Basemap from Matlab/Earthstar Geographics/Maxar).

As part of a broader effort to model future coastal hazards^23,24^ along the U.S. South Atlantic Coast (Miami, Florida to Cape Henlopen, Delaware, see Fig. 1A, which is roughly 1850 km), we applied CoSMoS-COAST, a data-assimilated, reduced-complexity shoreline model (detailed in Vitousek et al.^12,18,21,25^ and summarized in “Methods” section) to project long-term coastal change in the region. The model is comprised of 34,067 shore-perpendicular transects, with roughly 50 m spacing in the alongshore direction (see zoom near Cape Hatteras, North Carolina in Fig. 1B, for example). The model is forced with hindcasted and climate-model-projected wave and sea-level conditions (blue and orange lines on Fig. 1C, respectively) as detailed in “Methods” section. The model assimilates historical satellite-derived shoreline observations (blue dots + blue whiskers in Fig. 1D) produced for the region using the CoastSat toolbox^23,24,26^. The model provides an assessment of its parametric and structural uncertainty (i.e., pink and yellow bands on Fig. 1D, respectively, which are described in “Methods” section), which surround the model’s median projections (i.e., red line on Fig. 1D). After the model is calibrated (1990–2015) and validated (2015–2020), it is then run until 2100 to provide a projection of the future shoreline position and uncertainty along the scale of the U.S. South Atlantic Coast under different sea-level rise and model cases (see “Methods” section and S4).

**Fig. 2:**
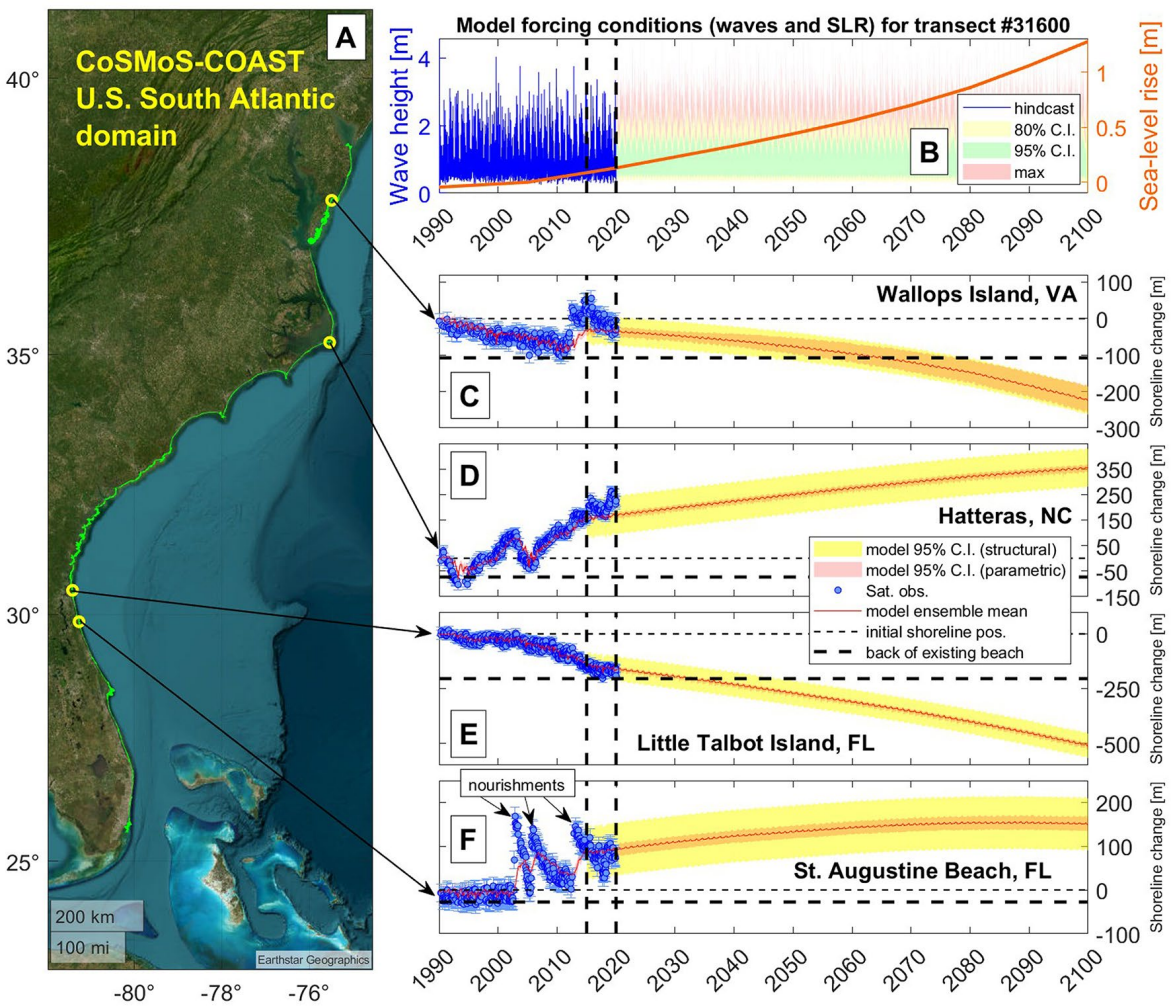
Examples of long-term simulations of shoreline change with CoSMoS-COAST until 2100. The extent of the U.S. South Atlantic Coast model domain (panel A—transects in green) with model results at Wallops Island, Va., Hatteras, N.C., Little Talbot Island, Fla., and St. Augustine, Fla. (transect # 31600, 25880, 11150, and 9805, respectively). Panel B shows hindcasted (blue line) and projected (ensemble) wave (green, yellow, and red confidence bands) and sea-level rise conditions (orange line) applied to the model at transect #31600 (Wallops Island, Va.). Panels C, D, E, and F show the long-term modeled shoreline position (red line) and its parametric (pink band) and structural uncertainty (yellow band). The parametric uncertainty (pink band) is calibrated (from 1990–2015) with satellite-derived shoreline observations (blue circles) and their confidence intervals (blue ‘whiskers’). Later in the simulation, the model is validated (during the period 2015–2020) to assess the model’s structural uncertainty ($\pm 2\varepsilon_{{{\text{RMSE}}}}$, where $\varepsilon_{{{\text{RMSE}}}}$ is the model root-mean square error (RMSE) calculated using Eq. (2) and is shown in yellow confidence bands), which is propagated forward with a constant width during the model ‘projection period’ (2020–2100). Note that anomalies in shoreline position due to beach nourishments (e.g., panel F), generally mask naturally occurring erosion trends and thus increase the tendency for the model to infer/assimilate accretion trends from historical observations. (Basemap from Matlab/Earthstar Geographics).

**Fig. 3:**
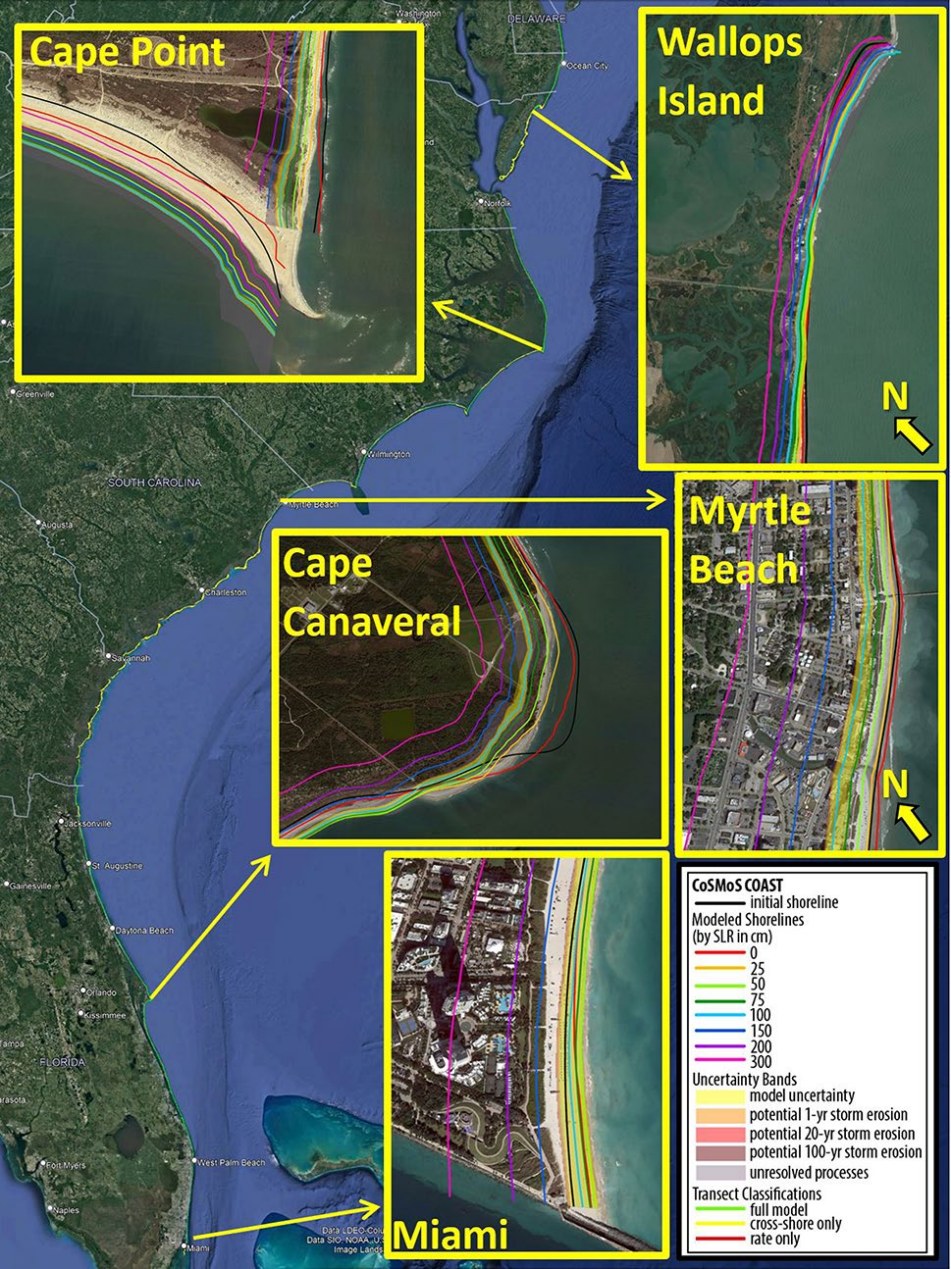
Examples of shoreline modeling projections for ~ 1,850 km of coastline in on the U.S. South Atlantic Coast (full data available online at [SC, NC], [FL, GA, VA, MD, DE]), which are shown here for the “unimpeded” and “continued accretion” model case scenarios and the intermediate transgression slope (described in “Methods” section and shown in S8, respectively). The projections represent the shoreline position in 2100 with various projections of sea-level rise. The yellow bands represent the projected shoreline position and (parametric) uncertainty, and the orange/red bands (shown only for the 1.0 m sea-level scenario for clarity) represent the potential storm-driven erosion uncertainty determined following Vitousek et al.^21^. (Basemap from Google Earth).

Figure 2 shows long-term projections of coastal change at a few transects along the U.S. South Atlantic Coast at Wallops Island, Va., Hatteras, N.C., Little Talbot Island, Fla., and St. Augustine, Fla. (which represent different types of coastal behavior in terms of accretion versus erosion and limited versus significant anthropogenic influence). Site-specific shoreline projections (see e.g., Figs. 2, 3, and the accompanying Data Releases [SC, NC], [FL, GA, VA, MD, DE] for the projections across the full region) are highly sensitive to local shoreline characteristics/trends and projections of relative sea-level rise. Some locations on the U.S. South Atlantic Coast (under certain sea-level scenarios) are projected to accrete in 2100 relatively to the current shoreline position (e.g., Fig. 2 panels D and F depicting Hatteras, N.C., and St. Augustine, Fla., respectively, as well as southern Cape Point in Fig. 3) and others are projected to erode (e.g., Fig. 2 panels C and E depicting Wallops Island, Va., and Little Talbot Island, Fla., respectively, as well as the Wallops Island, Myrtle Beach, Cape Canaveral, and Miami cut-outs in Fig. 3). In some locations, the long-term trajectory of shoreline change is highly uncertain, often due to episodic human interventions (e.g., beach nourishments, as shown in Fig. 2 panel F), which can affect historical shoreline positions and mask naturally occurring rates of erosion (as discussed below). Furthermore, the frequency, magnitude, and/or extent of human interventions may change in the future with changing oceanographic conditions, although this is not readily predictable nor is accounted for in the context of the current modeling system.

**Fig. 4:**
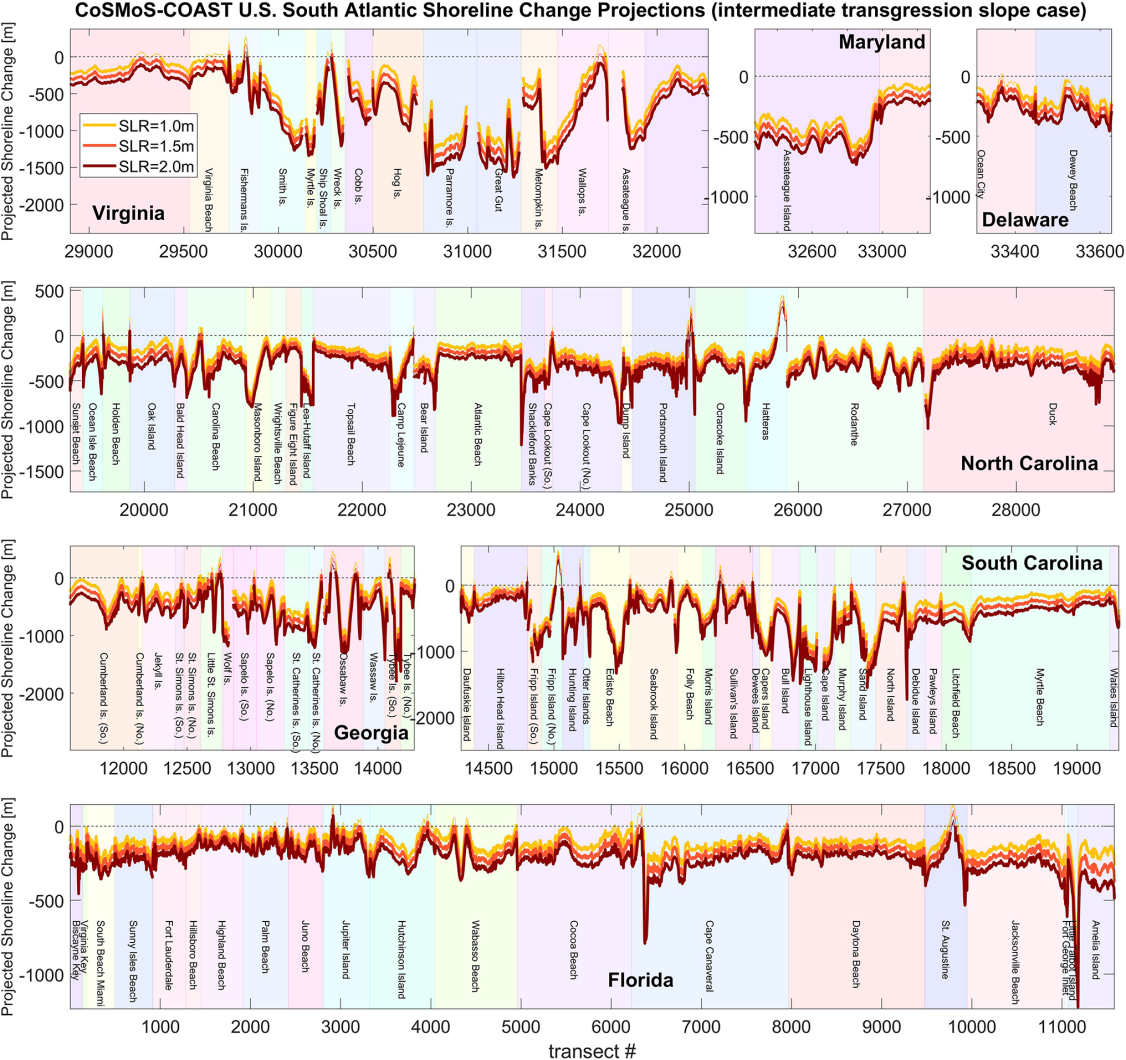
Model-projected shoreline positions in 2100 (relative to the initial shoreline position obtained from satellite-derived shoreline observations ca. 1990) versus transect number (numbered consecutively from south to north) for the intermediate transgression-slope scenario (see S10 and S11 for plots of the high and low transgression slope scenarios, respectively). Note that each panel represents a different state on the U.S. South Atlantic Coast, and each panel has different limits on the y-axis. The figure illustrates the shoreline-change simulations due to sea-level rise (SLR) projections of 1.0, 1.5, and 2.0 m by 2100 in yellow, orange, and red colors, respectively, under the “unimpeded” and “continued accretion” model cases (see “Methods” section). Sections of the yellow, orange, and red lines in this figure are plotted as either thin or thick lines, which indicate that the projected shoreline position is either seaward or landward of the existing end of the sandy beach, respectively. Hence, the thin line segments indicate portions of the coastline where sandy beaches are still present in 2100, whereas thick line segments indicate where sandy beaches have become lost in 2100, while assuming a static back beach line.

Figure 4 illustrates the extent of model-projected shoreline change in 2100 along the U.S. South Atlantic Coast (relative to the initial shoreline position obtained from satellite-derived observations ca. Jan. 1, 1990) due to 1.0, 1.5, and 2.0 m of sea-level rise by 2100, which are shown in yellow, orange, and red colors, respectively. The simulations, shown here, are based on the “unimpeded” and “continued accretion” model case scenarios and an intermediate transgression slope (described in “Methods” section and shown in S8, respectively). Figure 4 also shows several prominent littoral cells (i.e., explicitly identified groups of adjacent model transects separated by jetties, inlets, etc.) along the U.S. South Atlantic Coast, and we find that the largest amounts of change generally occur at the ends of littoral cells. This finding is consistent with historical shoreline trends (shown in S7) and the notion that jetties and inlets interrupt longshore transport, leading to the large gradients in longshore transport (see term [1] in Eq. (1) in “Methods” section, for example), and, consequently, the large rates of coastal change. We also find that small barrier islands along the coast of Georgia, South Carolina, and northern Virginia generally exhibit the largest degrees of erosion due to their dynamic nature (evidenced by their large historical shoreline trends, shown in S7) and their gentler transgression slopes (see S8), which control future-sea-level-driven recession (according to term [2] in Eq. (1) in “Methods” section). However, these coastal settings are also generally where the model is least accurate (see S6), which is likely due to the complexity of wave-driven cross-shore and longshore transport, wave shadowing, and inlet bypassing in the region^12,21,27^. The model does not account for varying rates of erosion through different substrates (e.g., hardened coastal infrastructure and vegetation) but instead treats the entire transect as a sandy substrate during the “unimpeded” model case (which is further described in “Methods” section). Thus, the results in Fig. 4 may represent an upper bound of potential erosion, relative to a real-world setting, but nevertheless may be appropriate for undeveloped, lightly vegetated environments. In hardened urban environments, potential erosion may likely resemble the “impeded” model case (also described in “Methods” section), which represents a lower bound of shoreline recession into back-beach environments.

Sections of the yellow, orange, and red lines, shown on Fig. 4, are plotted as either thin or thick lines, which indicate that the projected shoreline position is either seaward or landward, respectively, of the present-day landward end of the existing sandy beach. Hence, the thin line segments indicate portions of the coastline where sandy beaches are projected to be present in 2100, whereas thick line segments indicate portions of the coastline where sandy beaches are projected to erode to present-day landward beach boundaries by 2100. As shown in Fig. 4, there are very few locations where the shoreline is projected to accrete by 2100.

**Fig. 5:**
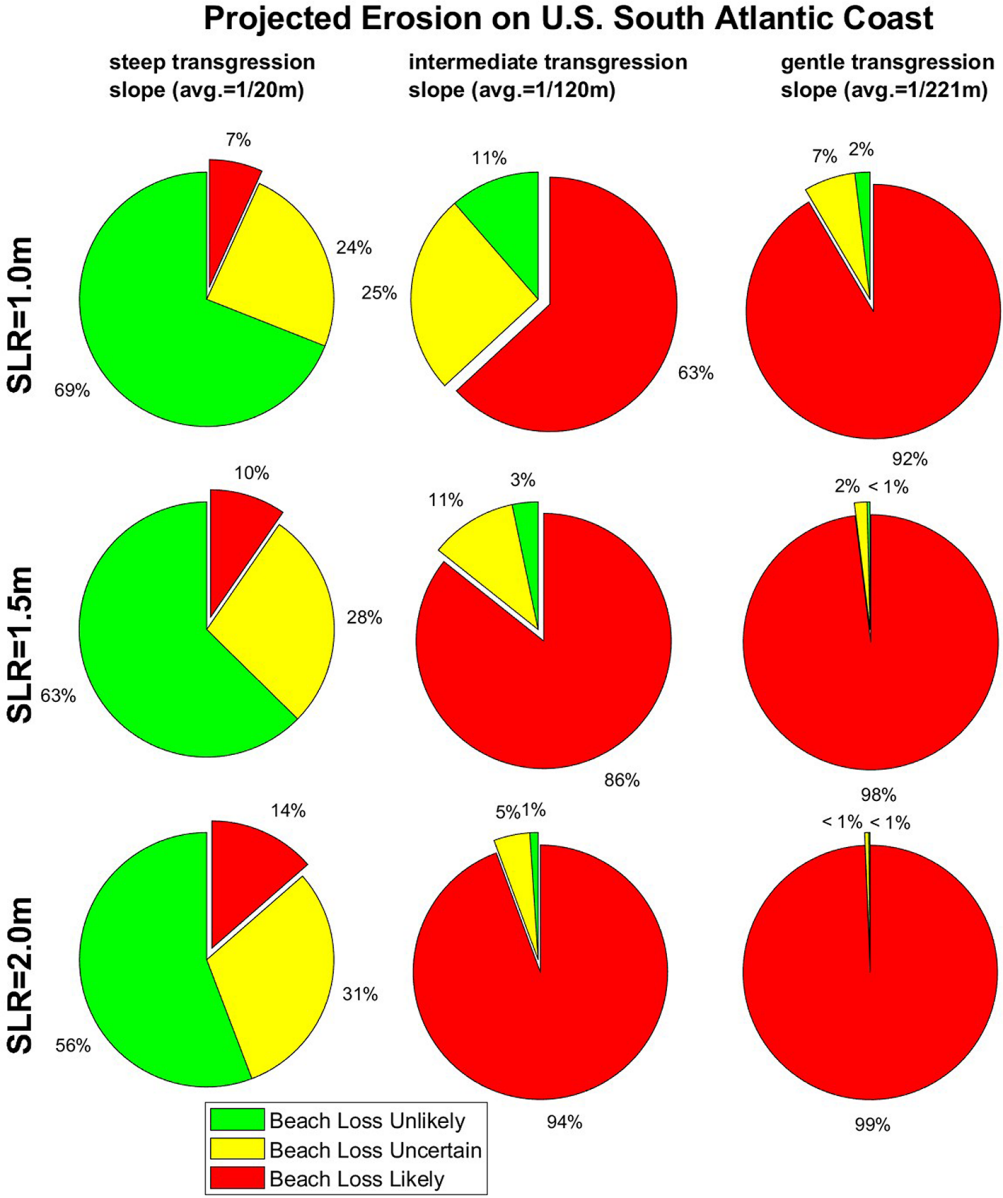
Projected beach loss on the U.S. South Atlantic Coast (Florida to Delaware) as a function of sea-level rise (i.e., different rows) and transgression-slope case (i.e., different columns), and assuming the “impeded” model case, i.e., hardened, unyielding back-beach infrastructure and vegetation, which represent lower-bounded projections of potential beach change. The green, yellow, and red sections of the pie charts indicate the percentage of coastline with low, intermediate, and high likelihood of beach loss (i.e., where the modeled shoreline recedes landward into existing beach boundaries of urban infrastructure or back-beach vegetation). Note that the prevalence of beach loss is highly sensitive to the transgression slopes applied to the model (which are spatially variable and are shown in S8).

The following analysis (shown in Fig. 5) explores the prevalence of projected beach loss (i.e., the thick lines plotted here) compared to beach persistence (i.e., the thin lines plotted here). We analyze the percentage of the U.S. South Atlantic Coast that is projected to erode past the present-day, landward end of sandy beaches under three sea-level scenarios (1.0, 1.5, and 2.0 m) with three different transgression slopes (shown in S8 and representing the steeper foreshore beach slope, the more gentle active beach profile slope, and the average of the two, which is called the intermediate case). Figure 5 categorizes the likelihood of beach loss as “beach loss unlikely” (where the projected shoreline position and the landward limit of its 95% (structural) uncertainty bounds remains seaward of the existing end of the sandy beach), “beach loss uncertain” (where the 95% uncertainty bands enclose the present-day division between sand and land), and “beach loss likely” (where the seaward limit of the 95% confidence bands has eroded past the present-day division between sand and land).

The results in Fig. 5 indicate that the prevalence of beach loss in “impeded” (i.e., hardened, unyielding) back-beach environments on the U.S. South Atlantic Coast increases with sea-level rise and with gentler transgression slopes, with transgression slopes seemingly representing the larger factor in future beach loss. For example, with 1.0 m of sea-level rise by 2100, the model projects that 7, 63, and 92% of the U.S. South Atlantic Coast will experience complete beach loss under the steep, intermediate, and gentle transgression slope cases, respectively (which correspond to 20, 120, and 221 m of sea-level driven recession, on average). On the other hand, applying the intermediate transgression slope case, the model projects that 63 to 94% of transects across the U.S. South Atlantic Coast will likely experience beach loss by 2100 under 1.0 to 2.0 m of sea-level rise, respectively.

## Discussion

By design, physics-based models excel at capturing physical processes and interactions that affect natural coastal and marine systems. However, they often cannot easily capture the effects of anthropogenic influences on these systems (e.g., beach nourishments). The inability to model anthropogenic influences on coastal systems is encapsulated in the paper “Is there a bulldozer in your model?” by Lazarus and Goldstein^28^. Unlike physics-based models, data-driven models can implicitly account for anthropogenic influences in the coastal zone, in so far as those anthropogenic influences are reflected in the shoreline observations used to drive the model. For example, there are numerous beach nourishments present in the satellite-derived shorelines observations on the U.S. South Atlantic Coast. In fact, the accretion spikes in 2017–2018 in Fig. 1D and in 2003, 2005, and 2013 in Fig. 2F are caused by beach nourishments. The model does not explicitly parameterize individual nourishment events and the presence of nourishments in the shoreline observations can lead to mismatches between the model (e.g., in red Figs. 1D and 2F) and the observations (in blue) when data assimilation is turned off during the validation period (2015–2020). The mismatch/error between model and observations subsequently increase the model’s structural uncertainty (yellow bands in Figs. 1, 2). Nevertheless, the modeling approach does capture (at least implicitly) the role of artificial beach nourishments in stabilizing naturally occurring erosion trends (and the expanded location-dependent uncertainty resulting from local nourishments) by the residual shoreline trend term (i.e., term [3] in Eq. (1) in “Methods” section), which is estimated via data assimilation. This suggests that the figurative ‘bulldozer’ (i.e., anthropogenic influence on shoreline behavior) is at least partially captured in the current hybrid model approach, presented here, alongside natural processes such as longshore and cross-shore sediment transport. Although the model does not explicitly identify, parameterize, and project individual nourishments and their impact on the future shoreline positions, such an endeavor may be valuable for future research on adaptation pathways. Nevertheless, the erosion projections presented here (e.g., Fig. 4) can help to understand beach volume deficit and guide estimates of nourishment volumes needed to maintain existing beach widths. For example, multiplying the projected erosion (on Fig. 4) by the approximate depth of closure (approximately 10 m) provides an estimate of the beach volume deficit per meter of beach in the alongshore direction. For Florida, where approximately 250 m of shoreline recession is projected by 2100 (under 2.0 m of sea-level rise with the intermediate transgression slope,see Fig. 4), this corresponds to needing approximately 2.5 million cubic meters of nourishment per kilometer of beach in order to maintain historical beach widths.

Sea-level driven recession and the human response to it are the greatest sources of uncertainty in long-term projections of shoreline change on the U.S. South Atlantic Coast, which is in agreement with many other studies^29–31^. In particular, the uncertainty related to the transgression slope, i.e., the ratio of sea-level rise to the resulting shoreline recession^13^^,^ which stems from application of the ‘Bruun rule’^32^, dominates the overall uncertainty in long-term ‘natural’ coastal recession. Although, the uncertainty related to the sea-level rise projections used here (1.0, 1.5, and 2.0 m by 2100) is not trivial, it is only responsible for a factor of 1.5–2 increase in shoreline recession (relative to the 1.0 m baseline case) among the most plausible sea-level scenarios considered here. On the other hand, the choice of applying the intermediate or gentle transgression slope rather than the steep transgression slope will increase the shoreline recession by a factor of 5–10 (or more, see S8). If higher rates of shoreline transgression ensue, then it is likely that human interventions (e.g., nourishment, armoring) will intensify in response, given that such interventions are already widespread across the region^33^.

By regressing rates of historical sea-level rise and long-term erosion rates on the Atlantic Coastline, Leatherman^34^ and Zhang et al.^35^ found average transgression slopes of 1/150 and 1/78, respectively. However, these approaches have received criticism^36,37^ over the validity of the stand-alone Bruun rule and the ambiguity of the site-selection process, since sites that are accreting (naturally or otherwise) and those with substantial longshore transport gradients (e.g., sites near the ends of a littoral cell) must be omitted from the analysis. A regional attempt to estimate the observed transgression slopes (from the long-term, historical sea-level rise rates and erosion rates derived from satellite shoreline observations (1990–2020) shown in S7) is given in S9. The average erosion rate is − 0.062 m/yr over the entire U.S. South Atlantic Coast (i.e., across the results in S7). Dividing this rate by a nominal rate of sea-level rise of 4 mm/yr (which closely resembles the sea-level trends observed via tide gauges across the U.S. South Atlantic Coast) gives a transgression slope of 1/15.5, which closely resembles that of the passive flooding case (i.e., the “steep” transgression slope case which has an average of 1/20). However, one can only look at the pronounced longshore variability in historical shoreline change rate (i.e., in S7) on the U.S. South Atlantic Coast (particularly the high incidence of accretionary beaches), to recognize that taking this simplified approach to estimating the transgression slope (i.e., S9) is indeed fraught with complications arising from beach nourishments and the end-effects of littoral cells. For example, in the current analysis, shorelines on the east coast of Florida appear to be accreting by 0.43 m/yr on average, while sea level has risen ~ 10 cm over the same period. However, this average accretion signal is largely due to the effects of beach nourishments masking natural signals of shoreline recession^33^, as discussed above. Furthermore, recent studies hypothesize a time lagged response of coastal barrier-island retreat to sea-level rise^38^, which is not compatible with previous attempts to estimate the transgression slope from recent historical observations. In short, we believe that attempts to understand sea-level-driven recession in field settings are hampered by small signal-to-noise ratios resulting from the large (and highly variable) rates of historical shoreline change compared to small (and relatively alongshore uniform) rates of historical sea-level rise, which precludes making credible estimates of transgression slope (e.g., S9) for such a coastal setting. This makes it rather difficult to observationally constrain (e.g., via data assimilation) the effects sea-level rise on long-term coastal recession in open coastal environments (i.e., term [3] in Eq. (1) in “Methods” section). On the other hand, we believe the data-assimilated modeling approach presented here is relatively well-suited to attribute historical shoreline change to longshore and cross-shore wave-driven sediment transport (and the interruption of these transports by littoral cells), as well as local shoreline trends (i.e., terms [1], [4] and [3], respectively, in Eq. (1) in “Methods” section).

Although field-scale validations of the Bruun rule remain elusive on open-ocean beaches, quantitative validation studies of the Bruun rule have recently been carried out in laboratory settings (e.g., Atkinson et al. 2018^39^) and in lakes where large water-level variations occur on decadal time scales. For example, Troy et al.^40^ and Abdelhady et al.^41^ found that observed transgression slopes (associated with > 1.0 m water-level variations over decadal time scales) generally fall in between values of the foreshore beach slope (or the passive-flooding beach slope) and the classic Bruun slope (i.e., the ratio of depth of closure plus the berm height to the horizontal distance between the two features). Across Abdelhady et al.’s^41^ eleven Lake Michigan sites, the median of the observed transgression slope was found to be approximately twice that of the foreshore beach slope and half that of the active beach profile (i.e., Bruun) slope (during a 1–2 m lake level rise event from 2013 to 2020). For beaches exposed to the open ocean, however, it remains unclear if the transgression slope scales with (1) the active beach profile slope (i.e., the classic Bruun rule), (2) the foreshore or inland beach slope (i.e., the passive inundation model), or (3) some landscape-dependent middle ground. Furthermore, the current model does not account for non-linear interaction effects between elevated waves and water levels and the resulting coastal erosion, although some theoretical frameworks that account for these interactions are emerging^42–44^. Nevertheless, the recent findings in the Great Lakes have motivated the current approach of applying a range of gentle, intermediate, and steep transgression slopes to the U.S. South Atlantic Coast.

When applying the intermediate transgression slopes and extrapolating present-day shoreline accretion trends (which potentially arise from beach nourishments, where they exist), the model projects that 63 to 94% of the shorelines on the U.S. South Atlantic Coast will retreat up to or past the present-day extent of sandy beach under 1.0 to 2.0 m of sea-level rise, respectively. Projections of 63 to 94% beach loss (assuming that the back-beach line is held) on the U.S. South Atlantic Coast appear to be more severe and widespread than the projections of 24 to 75% beach loss in California using the same data-assimilated modeling approach^21^. However, such differences between the vulnerability of the West Coast and East Coast of the U.S. seem to make sense given that they stem from the gentler transgression slopes that characterize the passive continental margin setting of the U.S. South Atlantic Coast relative to the active margin coast of California. Furthermore, the widespread coastal management practice of beach nourishments on the U.S. South Atlantic Coast (which are far more pervasive than on the U.S. West Coast) seem to be a forerunner of future challenges related to accelerated sea-level rise and coastal erosion.

One of the largest factors contributing to the uncertainty in the magnitude of transgression slopes is the interaction of shoreline, dune, vegetation, and overwash processes^5^. Dune/vegetation/overwash processes are not modeled explicitly in CoSMoS-COAST, but instead the co-evolution of dunes and vegetation with the shoreline (under the assumption of maintaining an equilibrium beach elevation profile) is lumped into the sea-level-driven, cross-shore recession term (i.e., term [2] in Eq. (1) in “Methods” section). There are, however, an increasing number of models that try to explicitly resolve shoreline, dune, vegetation, and overwash processes in physics-based models like XBeach^45^ and in reduced complexity-models like CASCADE^46–48^, BRIE^49^, and Barrier3D^50^. While physics-based morphologic models are becoming increasingly capable of simulating storm-driven water levels and erosion^9^, there are still rarely applied to long-term simulations^5^, primarily due to computational constraints (e.g., S3). Furthermore, physics-based coastal morphology models are seldom, if ever, compared against observations spanning more than a single storm event, which further highlights the benefits of the hybrid modeling approach presented here, despite its limitations. Future work might explore how storm-scale processes (wave-driven water levels, dune erosion and overwash) and associated parameters can be captured in hybrid or meta-modeling approaches^51^ and assimilated with satellite observations of multiple coastal-change features such as dune toe or vegetation lines^52^ in addition to the observed shoreline position^53^.

## Conclusions

Predicting natural coastal response to sea-level rise remains a grand-challenge problem in the field of coastal science. Prediction becomes further complicated in coastal environments with high anthropogenic influence. For both natural and anthropogenic coastal settings, a thoughtful synthesis of data and models is often needed to make reliable predictions.

Here, we have applied a large-scale, long-term, data-assimilated shoreline-change modeling system across ~ 1850 km of coastline on the U.S. South Atlantic Coast, home to a variety of natural and urbanized coastal geomorphic settings. Across the U.S. South Atlantic Coast and following the calibration (1990–2015) and validation periods (2015–2020), the model achieves an median root-mean square accuracy of $\varepsilon_{{{\text{RMSE}}}} = 12.3{\text{ m}}$ and an index of agreement of $d = {0}{\text{.44}}$, indicating that the model performs well over the region, despite some areas of poorer performance (e.g., small barrier islands). The model projections, although subjected to considerable uncertainty, indicate that significant coastal erosion may occur due to accelerated sea-level rise. Approximately 63 to 94% of the shorelines on the U.S. South Atlantic Coast are projected to retreat past the present-day extent of sandy beach under 1.0 to 2.0 m of sea-level rise, respectively, despite the extrapolation of historical, nourishment-influenced shoreline trends. The projections suggest that many beaches on the U.S. South Atlantic Coast will become lost against the urban hardscape, or alternatively may require substantially increased management efforts (e.g., beach nourishments, sand retention, armoring, dune restorations, or other engineering and nature-based solutions) in order to maintain existing beach widths and the many services they provide.

## Methods

### Modeling shoreline change on the U.S. South Atlantic Coast using CoSMoS-COAST

Long-term shoreline evolution on the U.S. South Atlantic Coast (Miami, Florida to Cape Henlopen, Delaware) is modeled with CoSMoS-COAST^12,18,21,25^. The model solves a one-dimensional (alongshore) conservation of sediment volume equation, which is given by:

1 $$\underbrace {{\frac{\partial Y}{{\partial t}}}}_{{{\text{shoreline}}\;{\text{change}}}} = \overbrace {{\underbrace {{ - \frac{1}{{d_{c} }}\frac{\partial Q}{{\partial X}}}}_{{[1]{\text{ longshore}}\;{\text{transport}}}} - \underbrace {{\frac{c}{\tan \beta }\frac{\partial S}{{\partial t}}}}_{\begin{subarray}{l} [2]{\text{ shoreline}}\;{\text{migration}} \\ {\text{due}}\;{\text{to}}\;{\text{sea - level}}\;{\text{rise}} \end{subarray} } + \underbrace {{v_{lt} }}_{\begin{subarray}{l} [3]{\text{ long - term}}\;{\text{residual}}\;{\text{shoreline}}\;{\text{trend}}; \\ {\text{unresolved}}\;{\text{processes}} \end{subarray} }}}^{{{\text{long - term}}\;{\text{processes}}}} + \overbrace {{\underbrace {{\frac{1}{\tau }\left( {Y_{eq} - Y} \right)}}_{{[4]{\text{ cross - shore}}\;'{\text{equlibirum}}'\;{\text{transport}}}} + \underbrace {\varepsilon }_{{[5]{\text{ additive}}\;{\text{noise}}}}}}^{{{\text{short - term}}\;{\text{processes}}}}$$

As discussed in Vitousek et al.^21^, Eq. (1) synthesizes popular individual-process models including: (1) a ‘one-line’ model for longshore transport, (2) a cross-shore beach profile recession model due to sea-level rise, (3) a long-term residual shoreline trend that represents sediment sources and sinks, (4) a wave-driven cross-shore equilibrium shoreline model, and finally (5) a (Gaussian) noise term.

The U.S. South Atlantic Coast model is comprised of roughly 34,000 transects, spaced every 50 m in the alongshore direction. The model is forced with hindcasted and forecasted wave conditions obtained from the ERA5 reanalysis^54^ and seven CMIP6-forced WAVEWATCHIII models chosen from the HighResMip collection^55,56^, respectively. Time-dependent projections of future relative sea-level rise (including local vertical land motion observed at tide gauge sites) come from the NOAA 2022 Sea Level Rise Technical Report^57^, and a nearest-neighbor interpolation approach is used to map sea-level projections at each tide gauge to each model transect. The U.S. South Atlantic Coast model applies scenarios of 0, 0.25, 0.5, 0.75, 1.0, 1.5, 2.0, or 3.0 m of sea-level rise (S5). The sea-level scenarios greater than or equal to 1.0 m occur at 2100 and have their own curves versus time as shown in S5. On the other hand, sea-level scenarios of less than 1.0 m occur before 2100 (and are based on the 1.0 m sea-level curve, as shown in the light blue line in S5). In the main text, above, we focus on presenting scenarios of 1.0, 1.5, and 2.0 m of sea-level rise, which are considered more plausible in the latest NOAA report^57^. However, results for all sea-level scenarios are available in the accompanying Data Releases ([SC, NC], [FL, GA, VA, MD, DE]). As in Vitousek et al.^12,21^, the model considers two binary (i.e., on or off) coastal-management scenarios: (1) ‘impeded’ versus ‘unimpeded’ coastal-recession scenarios, which prohibit or allow the modeled shoreline to erode past a so-called ‘landward model boundary’ that delineates the present-day location that separates sandy beach from of non-sandy substrates such as infrastructure, vegetation, or coastal bluffs, and (2) ‘continued accretion’ versus ‘no continued accretion’ scenarios, which allow or prevent the persistence of residual accretion trends ($v_{{{\text{lt}}}} > 0$), respectively, that are often associated with beach nourishment. The model is also run with three different transgression slopes, i.e., steep, gentle, and intermediate, which are derived using coastal elevation-profile data^58^ of foreshore beach slope, nearshore beach slope to the depth of closure, and the average of the two, respectively, across the U.S. South Atlantic Coast model domain (as detailed and depicted in S8).

The model is calibrated via a 200-member, littoral-cell-based ensemble Kalman filter^21^ and 25 years of satellite-derived, historical shorelines observations obtained using the CoastSat toolbox^26^ (which are available via an the accompanying Data Releases [SC, NC], [FL, GA, VA, MD, DE]; Barnard et al.^23,24^. The CoastSat algorithm derives shoreline position from historical satellite imagery (in this case, from Landsat 1984–2020 and spanning the entire U.S. South Atlantic Coast model domain) using the marching-squares algorithm^59^ that contours the threshold of the Modified Normalized Difference Water Index (MNDWI) that optimally splits the image-segmentation classes of ‘water’ and ‘sand’ using Otsu’s^60^ method. CoastSat-derived shoreline observations have been validated against traditional shoreline surveys (e.g., GPS surveys) in a variety of coastal settings worldwide including Truc Vert, France,Moruya and Narrabeen-Collaroy, Australia,Tairua, New Zealand,Torrey Pines [San Diego, California], Ocean Beach [San Francisco, California], and Duck, [North Carolina] in the United States, providing typical root-mean-square accuracies on the order of 7–14 m^26,61^.

After the model calibration period (1990–2015), we validate the model by comparing model-predicted shoreline positions against satellite-derived shorelines observations (from 2015–2020) using two popular metrics. Firstly, we apply the root-mean-square error (RMSE) metric, which is given by:

2 $$\varepsilon_{{{\text{RMSE}}}} = \sqrt {\frac{1}{N}\sum\limits_{i = 1}^{N} {\left( {\left( {Y_{{{\text{obs}}}} } \right)_{i} - \left( {Y_{{{\text{mod}}}} } \right)_{i} } \right)^{2} } }$$

where $Y_{{{\text{mod}}}}$ and $Y_{{{\text{obs}}}}$ are the modeled and observed shoreline positions, respectively, among a time series of $N$ data.

Secondly, we apply the index of agreement metric^62^, which is given by:

3 $$d = 1 - \frac{{\sum\limits_{i = 1}^{N} {\left( {\left( {Y_{{{\text{mod}}}} } \right)_{i} - \left( {Y_{{{\text{obs}}}} } \right)_{i} } \right)^{2} } }}{{\sum\limits_{i = 1}^{N} {\left( {\left| {\left( {Y_{{{\text{mod}}}} } \right)_{i} - \overline{{\left( {Y_{{{\text{obs}}}} } \right)_{i} }} } \right| + \left| {\left( {Y_{{{\text{obs}}}} } \right)_{i} - \overline{{\left( {Y_{{{\text{obs}}}} } \right)_{i} }} } \right|} \right)^{2} } }}$$

where the overbar indicates the mean of a quantity. The index of agreement ranges from $0 \le d \le 1$ , with values close to zero and unity indicating poor and excellent performance, respectively. The validation period assesses that the model achieves a median root-mean-square accuracy of $\varepsilon_{{{\text{RMSE}}}} = 12.3{\text{ m}}$ and an index of agreement of $d = {0}{\text{.44}}$ across the entire U.S. South Atlantic Coast domain (see S6 for depictions of spatial variability in model accuracy). The U.S. South Atlantic Coast model, presented here, has a slightly lower performance than the California Model presented in Vitousek et al.^53^ (i.e., and average index of agreement of 0.44 for the U.S. South Atlantic Coast vs. 0.56 in California). This is likely due to the prevalence of seasonal shoreline variations (in response to seasonally consistent wave patters) in California, which are very well modeled by the wave-driven, cross-shore equilibrium shoreline model component (i.e., term 4 in Eq. (1)), but are not as prevalent in shoreline observations on the U.S. South Atlantic Coast^63^.

The poorest model performance (i.e., the highest RMSE and the lowest index of agreement) generally occurs at the ends of small barrier islands, which predominantly occur in Georgia (GA), South Carolina (SC), and northern Virginia (VA) (red and purple markers in S6). The ends of small barrier islands generally exhibit the largest rates of shoreline change on the U.S. South Atlantic Coast (see S7), likely due to complex inlet sediment-supply dynamics, resulting from patterns of tidal currents, wave transformation, and longshore sediment transport, which can exhibit significant interannual variability and are not well resolved in CoSMoS-COAST when treated as a bulk residual trend (i.e., via term [3] in Eq. (1)). Combining a 1-D, free-form shoreline model^48^ with an inlet migration model^49,64,65^ or accounting for the presence of coastal dunes^46,50^ may improve shoreline predictability for barrier islands. Alternatively, regions on the U.S. South Atlantic Coast of poor performance with CoSMoS-COAST may indicate that more complex 2-D and 3-D modeling approaches may be needed to accurately predict the migration of small barrier islands and the inlets separating them.

To report regions of limited accuracy, the data-integrated CoSMoS-COAST framework (and the Data Releases [SC, NC], [FL, GA, VA, MD, DE] that accompany the model) provides an assessment of the model’s parametric and structural uncertainty (i.e., the sensitivity to unknown model parameters and the mismatch between model and observations, respectively, see Murray et al.^66^. The parametric uncertainty is propagated directly by the model (see Vitousek et al. 2021^18^), and the structural uncertainty is calculated as $\pm 2\varepsilon_{{{\text{RMSE}}}}$ confidence bands surrounding the model’s median projections, which seeks to contain ~ 95% of the possible variations/error, following Taylor and Kuyatt^67^. An example of the model’s parametric and structural uncertainty can be seen in the pink and yellow bands in Fig. 1D, respectively. In short, even in areas where model performance is relatively limited, the 1-D, data-integrated, ensemble modeling approach discussed here allows for an efficient yet robust assessment of potential future shoreline change with quantified uncertainty.

## Supplementary Information

Supplementary Information.

## Data Availability

The data [10.5066/P9W91314, 10.5066/P9BQQTCI)]^23,24^ produced and the model (https://doi.org/10.5066/P95T9188) used in this study are available online.
