# Supplementary material for: Scalable, data-assimilated models predict large-scale shoreline response to waves and sea-level rise
Source: Sci Rep. 2024 Nov 14;14:28029. doi: 10.1038/s41598-024-77030-4 (PMC11564573; doi:10.1038/s41598-024-77030-4)
Supplement: Supplementary file 1 — Supplementary Information. [file 41598_2024_77030_MOESM1_ESM.doc]

**Supplementary Material for “Scalable, data-assimilated models predict large-scale shoreline response to waves and sea-level rise”**

***Comparing computational effort of idealized 1-D, 2-D, and 3-D coastal models for long-term morphodynamic simulations***

We compare idealized estimates of computational work effort (based on Eq. , below) between 1-D (transect-based) and 2-D/3-D (grid-based) models of large-scale (i.e., 1,000 km), long-term (i.e., 100 year) coastal change with characteristic fine-scale grid sizes and time steps (given in , below). The results of the hypothetical ‘large-scale, long-term’ simulations (exemplified in and S3) indicate that 2-D and 3-D simulations can be approximately 72,000 and 43,200,000 times more expensive than the 1-D transect-based simulation. As demonstrated in S4, simulating 1 year [100 years] of coastal change across the ~34,000 transects of the U.S. South Atlantic Coast domain with 200 ensembles requires roughly 13 minutes [21.6 hours]. Assuming the factors of increase in computational burden reported in in Methods apply, then idealized 2-D and 3-D models would require roughly 178 and 106,520 years, respectively, without any parallelism. Alternatively, parallel simulations of the idealized 2-D and 3-D models would require roughly 1.78 and 213 years with 100x and 500x parallel speedup across smaller model subdomains, respectively. Hence, to achieve any parity in terms of computationally efficiency with an idealized 1-D model, the 2-D and 3-D models would require parallelism across tens of thousands to millions of computational units. Although this degree of parallelism is possible (see e.g., Dietrich et al., 2012), it is not trivial to achieve.

Thus, the assessment of potential computational effort between idealized 1-D, 2-D, and 3-D models, presented here, indicates that, for all intents and purposes, simulations of large-scale, long-term coastal-change demand the use of reduced-complexity or hybrid models (as least within the current state of the art), like the model described below.

***Methods for comparing computational effort of idealized 1-D, 2-D, and 3-D coastal models for long-term morphodynamic simulations***

To estimate the total computational work effort, $E$, required by an idealized numerical model, we apply the following equation:

$E\text{ \textasciitilde}\underbrace{\left(N\right)^{p}}_{\text{[1] spatial resolution}}\times\underbrace{\frac{t_{\text{sim}}}{\Delta t}}_{\text{[2] time stepping}}\times\underbrace{N_{\text{ens}}}_{\text{[3] \# of ensembles}}$ .

$E$ is the product of three dimensionless numbers, which represent [1] the number of grid cells, $N$, scaled by the algorithmic complexity exponent, $p$, [2] the number of time steps (where $t_{\text{sim}}$ and $\Delta t$ represent the total simulation time and the time step, respectively) and [3] the number of ensembles $N_{\text{ens}}$, respectively. Note that $E$ is written in terms of proportionality to the terms on the right-hand side because, in practice, the precise computational work effort (in terms of the total number of floating-point operations on a computer) will depend on the details of the model implementation, e.g., the number of model state variables, the numerical methods/algorithms used, and the code optimization, which are not explored here.

In Eq. , the computational effort $E$ scales linearly with terms [2] and [3], meaning that either doubling the simulation time, halving the time step, or doubling the number ensembles will effectively double the computational effort. The spatial resolution contribution (i.e., term [1]), however, may not scale linearly with the number of grid points $N$. Many numerical models with explicit time-stepping methods will scale linearly (i.e., $p=1$) with $N$; however, models with implicit time-stepping methods can often scale poorly with$p=3$, since they generally require matrix inversions (see e.g., Moin 2010). Although explicit time-stepping methods offer some apparent scalability advantages, they often require drastically smaller time steps compared to implicit methods in order to maintain numerical stability, which can lessen their initially perceived advantages. In the current thought experiment, we assume that all the models scale linearly (i.e., $p=1$). However, in practice, the limited scalability of 2-D/3-D physics-based models relative to reduced-complexity/hybrid models may further exacerbate their relative computational burden beyond the idealized cases explored here.

The number of model grid points $N$ is a critical factor in assessing the model’s computational tractability. The smallest spatial scale resolved in a model is generally proportional to $\Delta x$. For example, $2\Delta x$ sets the minimum wavelength resolved in the model. The maximum resolved length scale is related to $L$, the size of the model domain. The ratio between the largest and smallest resolved scales, $N\sim L/\Delta x$, determines the number of model grid points. In 2-D, the number of grid points is approximately $N\approx\left(\frac{L}{\Delta x}\right)^{2}$ , and in 3-D the number is $N\approx\left(\frac{L}{\Delta x}\right)^{2}\left(\frac{H_{0}}{\Delta z}\right)$ , where $\frac{H_{0}}{\Delta z}$ is the number of grid points (or “layers”) in the vertical direction (for example, $H_{0}$ is the average vertical scale (e.g., depth) and $\Delta z$ is the vertical grid-layer height). Likewise, the ratio between the longest resolved time scale, $t_{\text{sim}}$, and the smallest resolved time scale, $\Delta t$, determines the number of simulation time steps (i.e., term [2] in Eq. ). Finally, the number of ensembles $N_{\text{ens}}$(i.e., term [3] in Eq. ) is also an important factor in overall computational expense of a modeling exercise. Generally, the lesser the computational burden of an individual simulation, the more amenable the model is to being run in an ensemble, and vice versa (see e.g., Vitousek et al., 2021). Hence, 2-D and 3-D physics-based models are seldom run in large ensembles, in contrast to reduced-complexity models for which ensemble simulations are becoming ubiquitous.

|  | **grid length** (alongshore direction) | **grid spacing** (alongshore direction) | **grid length** (cross-shore direction) | **grid spacing** (cross-shore direction) | **grid length** (vertical direction) | **grid spacing** (vertical direction) | **simulation duration** (e.g., years of simulation) | **time step** |
| --- | --- | --- | --- | --- | --- | --- | --- | --- |
| 1-D (transect-based) coastal model | 1,000 km | 50 m | N\A | N\A | N\A | N\A | 100 years | 1 day |
| 2-D (medium resolution) coastal/ocean model | 10 km | 10 m | 10 km | 10 m | N\A | N\A | 100 years | 1 min |
| 3-D (high resolution) coastal/ocean model | 2 km | 2 m | 2 km | 2 m | 10 m | 1 m | 100 years | 5 sec |

S1 – Model grid & time-stepping parameters for simulations of long-term coastal change.

shows characteristic model grid and time-stepping parameters for long-term (i.e., 100 year) simulations of coastal change using 1-D (transect-based) and 2-D/3-D (grid-based) models. The characteristic model parameters in are used (via Eq. ) to estimate the relative computational costs of the 1-D, 2-D, and 3-D modeling approaches. Note that in , the grid resolution varies from ~ 50 m spaced transects to 10 m grid cells to 2 m grid cells for 1-D transect-based models, medium-resolution 2-D models, and high-resolution 3-D models, respectively. We note that 2-D and 3-D models, which represent coastal topography using raster-based (i.e., “stair-cased”) grids, generally require higher horizontal resolutions to adequately resolve vector-based representations of coastal features (e.g., shoreline position) that 1-D (transect-based) models can provide directly. The time steps for 1-D, 2-D, and 3-D models (shown in the last column of ) also differ significantly for each grid resolution due to numerical stability constraints, which are typically limited by the Courant number associated with resolving surface gravity waves, advection of momentum, or wetting and drying, depending on the numerical method (see e.g., Roelvink 2011).

compares idealized estimates of computational work effort (based on Eq. in Methods) between 1-D (transect-based) and 2-D/3-D (grid-based) models of large-scale (i.e., 1,000 km), long-term (i.e., 100 year) coastal change with characteristic grid sizes and time steps (given in ) needed to yield fine-scale results (i.e., every 10s to 100s of meters). The hypothetical ‘large-scale, long-term’ simulations presented in are intended to cover 1,000 km of coastline, but, for this thought experiment, the 2-D and 3-D simulation examples would, in practice, generally avoid using a single monolithic grid to model the entire coastline. Instead, we assume that the 2-D simulations are divided into 100 subdomains each with 10 x 10 m grid cells spanning 10 x 10 km areas. Likewise, the idealized 3-D simulations are assumed to use 500 subdomains each with 2 x 2 m grid cells spanning 2 x 2 km areas. The 1-D simulation, on the other hand, could easily span 1,000 km of coastline with a single domain of ~20,000 shore-normal transects with 50 m (or finer) spacing, like in the U.S. South Atlantic Coast case study, presented here.

S2 – Comparison of computational effort between 1-D, 2-D, and 3-D coastal models for

large-scale (>1,000 km), long-term (10-100 year) simulations.

|  | **subdomains** | **grid cells** (alongshore direction) | **grid cells** (cross-shore direction) | **grid cells** (vertical direction) | **total grid cells**  (for each subdomain) | **total grid cells**  (across all subdomains; in thousands) | **time steps**  (in thousands) | **ensembles** | **total work effort**  (in billions) | **factor of increase** (relative to 1-D model) |
| --- | --- | --- | --- | --- | --- | --- | --- | --- | --- | --- |
| **1-D** coastal model | 1 | 20,000 | N\A | N\A | 20,000 | 20 | 36.5 | 100 | **73** | **1** |
| **2-D** coastal/ocean model | 100 | 1,000 | 1,000 | N\A | 1,000,000 | 100,000 | 52,560 | 1 | **5,256,000** | **72,000** |
| **3-D** coastal/ocean model | 500 | 1,000 | 1,000 | 10 | 10,000,000 | 5,000,000 | 630,720 | 1 | **3,153,600,000** | **43,200,000** |

The hypothetical simulations presented in indicate that the idealized 2-D and 3-D simulations are approximately 72,000 and 43,200,000 times more expensive than the 1-D transect-based simulation. Furthermore, any sort of computational parallelism applied to this contrived modeling exercise would likely accomplish rather little. For example, assuming that the 2-D and 3-D models could be trivially parallelized by running each of the subdomains independently on separate computational nodes, then this would only save a factor of 100 and 500, respectively, which corresponds to the number of subdomains listed in .

The greatest advantage of 1-D coastal models is their linear scalability with the number of transects. For example, as shown in S4, CoSMoS-COAST scales linearly with the number of transects and the number of ensembles. In other words, doubling the alongshore resolution (i.e., the number of transects) doubles the overall computational effort. Furthermore, the use of data assimilation (i.e., the ensemble Kalman filter method described below) only increases the computational effort of CoSMoS-COAST by about 10% (see S4).

Unlike 1-D models, 2-D and 3-D models generally demand that cross-shore resolution increases in tandem with alongshore resolution, which causes the number of grid points to scale exponentially for 2-D and 3-D models as spatial resolution increases while grid cell aspect ratios (i.e., use of roughly square or cubic grid cells) are maintained. Further, as discussed above, numerical stability constraints often demand increasingly smaller time steps as spatial resolution is increased, which can further reduce computational scalability compared to the straightforward 1-D case, as presented in Section 2, above. In summary, we argue that the 3-7 orders of magnitude increase in computation cost (S3) will generally preclude the simulation of large-scale, long-term coastal change with traditional 2-D and 3-D physics-based hydrodynamic and sediment-transport models.

**Supplementary Figures:**

**
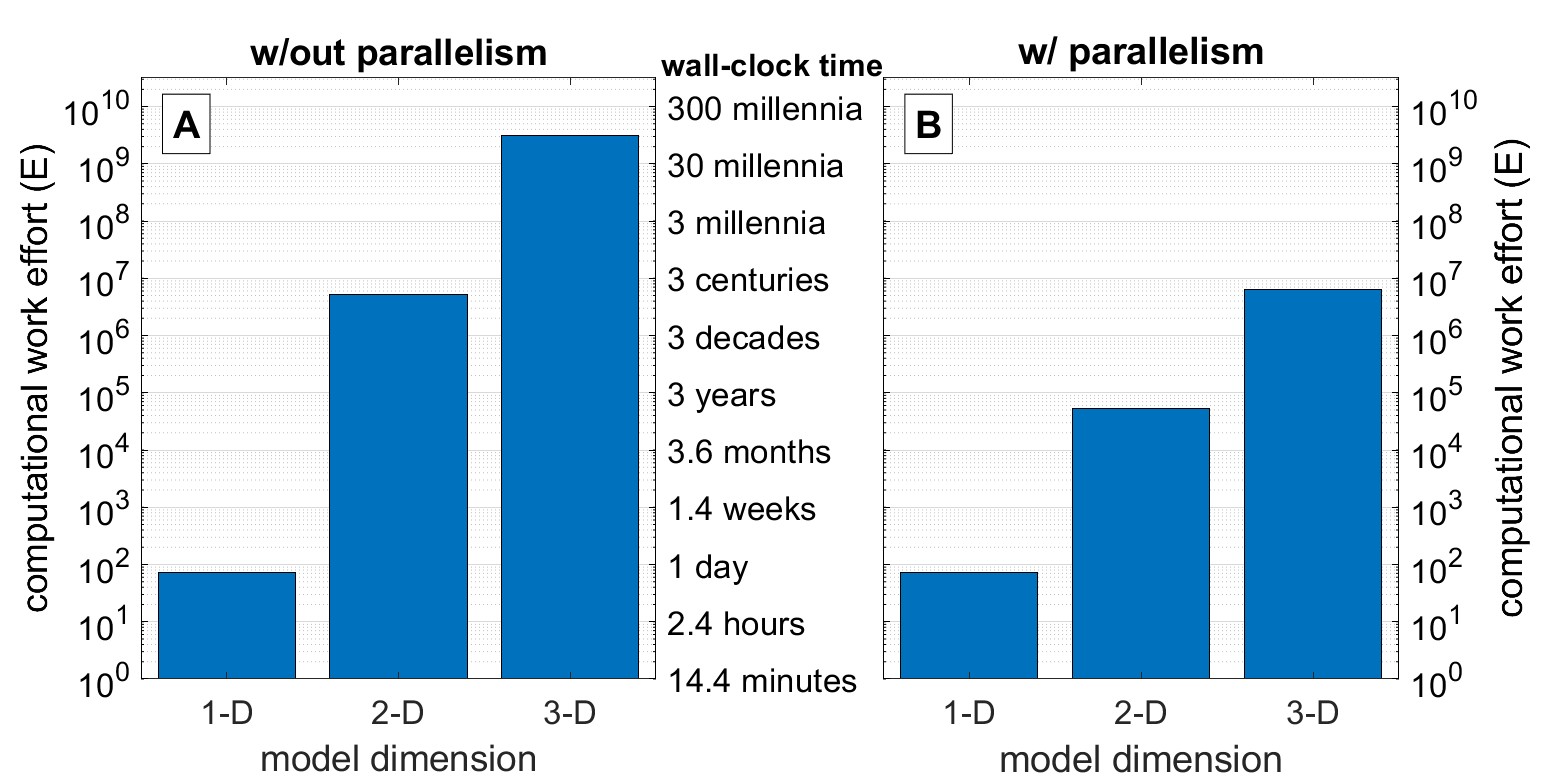
**

S3 - The approximate computational work effort ($E$, given in Eq. ; outer y-axes) and corresponding wall-clock time (central y-axis) for simulating large-scale, long-term coastal change, which are based on the idealized model parameters given in and for 1-D versus 2-D versus 3-D models. The figure indicates that 2-D and 3-D models are approximately 5 and 7 orders of magnitude more expensive, respectively, than 1-D models without parallelism (panel A). With 100x and 500x parallel speedup, 2-D and 3-D models are approximately 3 and 5 orders of magnitude more expensive, respectively, than 1-D models (panel B). The increase in computational cost of 2-D and 3-D models relative to 1-D models has effectively precluded their use for large-scale, long-term simulations of coastal change since such simulations would require approximately 2 to 200 years of ‘wall-clock time’ with modest parallelism.


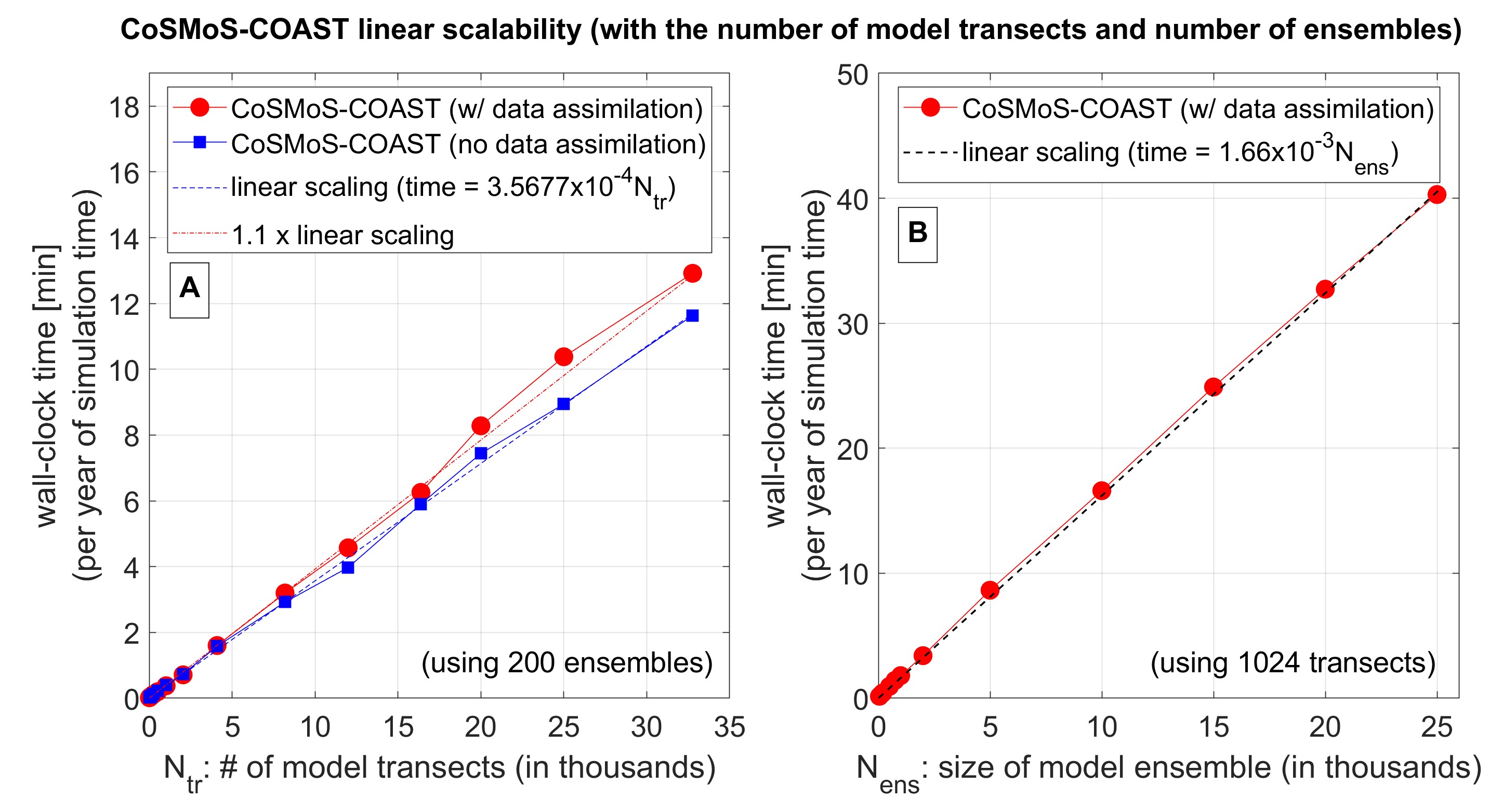


S4 - Computational time of the CoSMoS-COAST U.S. South Atlantic Coast model (with and without data assimilation shown in red and blue lines, respectively) as a function of the number of transects (on the x-axis in panel A) and the number of ensembles (on the x-axis in panel B). The results are obtained on desktop workstation using a single core of an Intel 12900K processor with DDR5-5200MHz RAM. The dashed lines represent linear scaling (i.e., doubling the number of transects (or doubling the number ensembles) doubles the computational cost). On panel A, the dashed red line represents 1.1x the linear function given in the dashed blue line, indicating that the use of data assimilation increases the overall computational effort by only 10%. The linear scaling efficiency of CoSMoS-COAST is notable because traditional 2-D sediment transport models generally do not possess linear scalability nor do they generally assimilate morphologic data (e.g., historical shoreline observations) (Any use of trade, firm, or product names is for descriptive purposes only and does not imply endorsement by the U.S. Government).


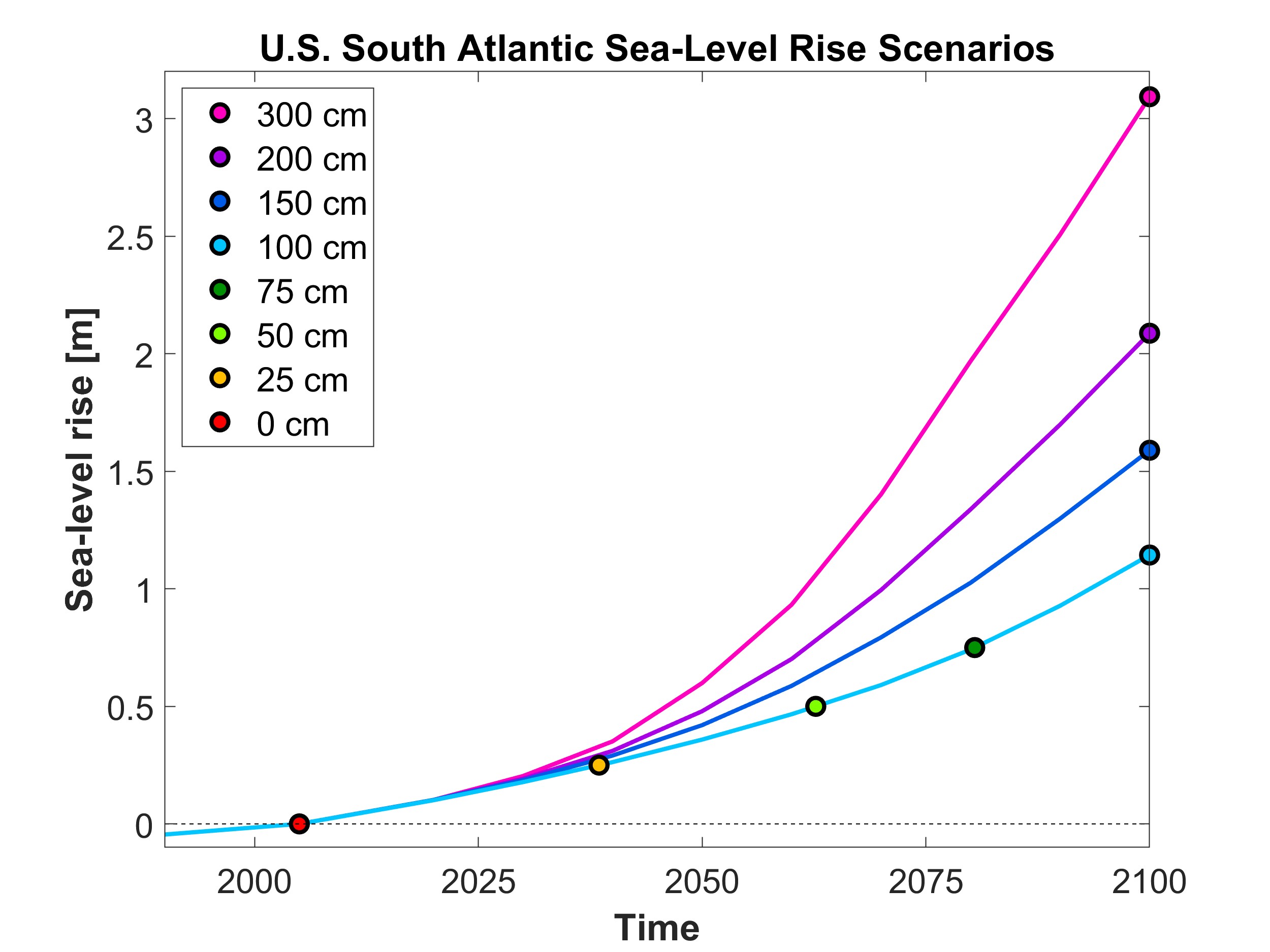


S5 - Scenarios of sea-level rise (i.e., 0, 0.25, 0.5, 0.75, 1.0, 1.5, 2.0, or 3.0 m) used in the current application are derived from the NOAA 2022 Sea Level Rise Technical Report (Sweet et al., 2022). Note that the sea-level rise scenarios that are less than 1.0 m of sea-level rise occur before 2100 (and are based on the 1.0 m curve – shown in the light blue line), while all scenarios greater than or equal to 1.0 m occur at 2100 and have their own curves with respect to time. Also note that even though they are referred to as the ‘1.0, 1.5, 2.0, or 3.0 m’ sea-level scenarios, their actual projected values by 2100 (obtained from the NOAA Report and which vary in space for each output location) may differ slightly from the evenly marked values mentioned in the manuscript.


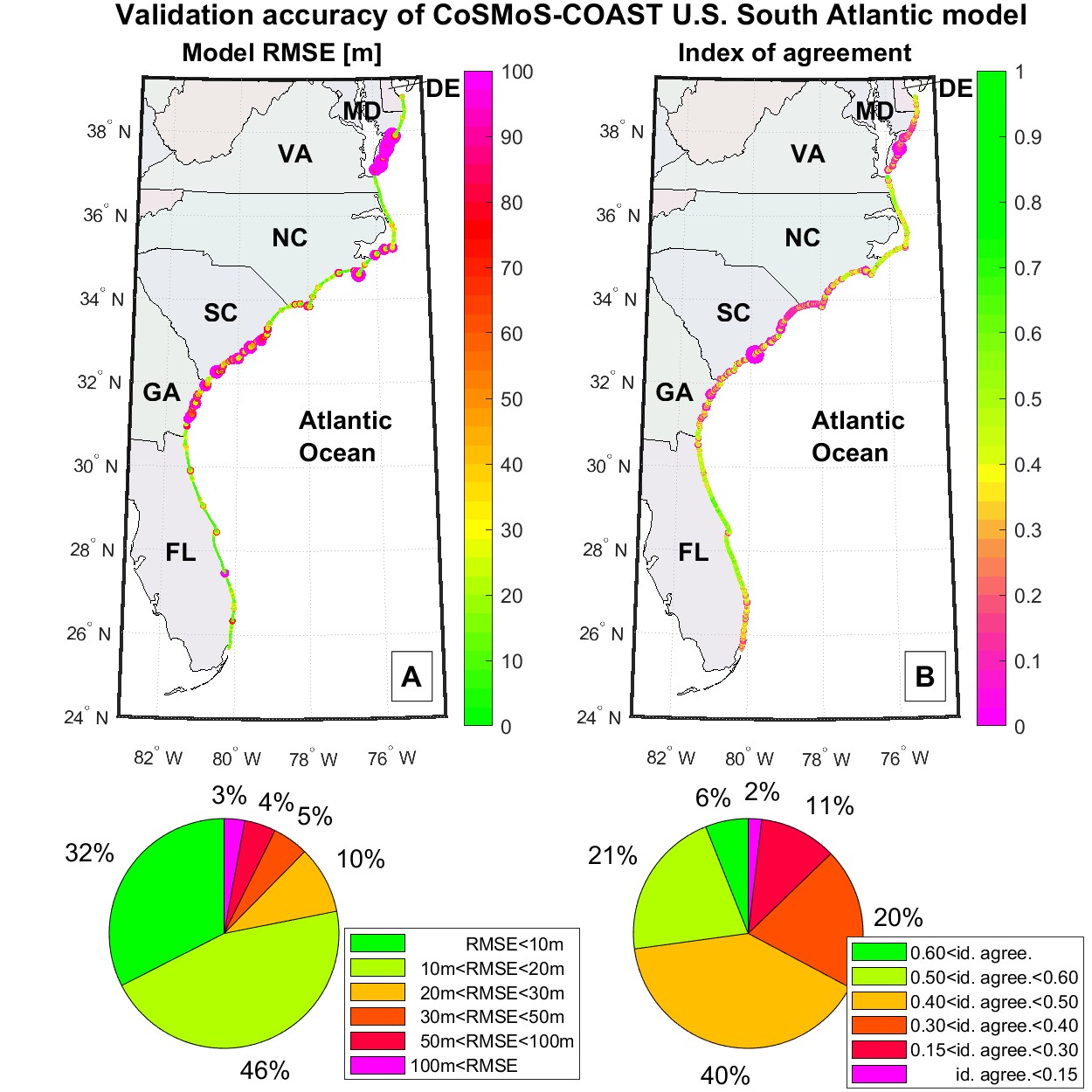


S6 - Spatial variability in CoSMoS-COAST model performance during the validation period (2015-2020) across the U.S. South Atlantic Coast domain. Panels A and B depict the model’s root-mean-square error (RMSE) (Eq. 2 in Methods) and index of agreement (Eq. 3 in Methods), respectively, between the model and satellite-derived shoreline observations with larger circles indicating poorer model performance. The bottom panels show pie charts (for each of the metrics shown above), which indicate various categories of model performance and their associated percentages across the entire U.S. South Atlantic Coast model domain. The model achieves a median root-mean-square accuracy of $\varepsilon_{\text{RMSE}}=12.3\text{ m}$ and an index of agreement of $d=0.44$ across the entire U.S. South Atlantic Coast domain during the validation period. However, the maps, shown here, indicate regions of poor model performance (i.e., the high RMSE and the low index of agreement), which often occur at small barrier islands, predominantly in Georgia (GA), South Carolina (SC), and northern Virginia (VA).


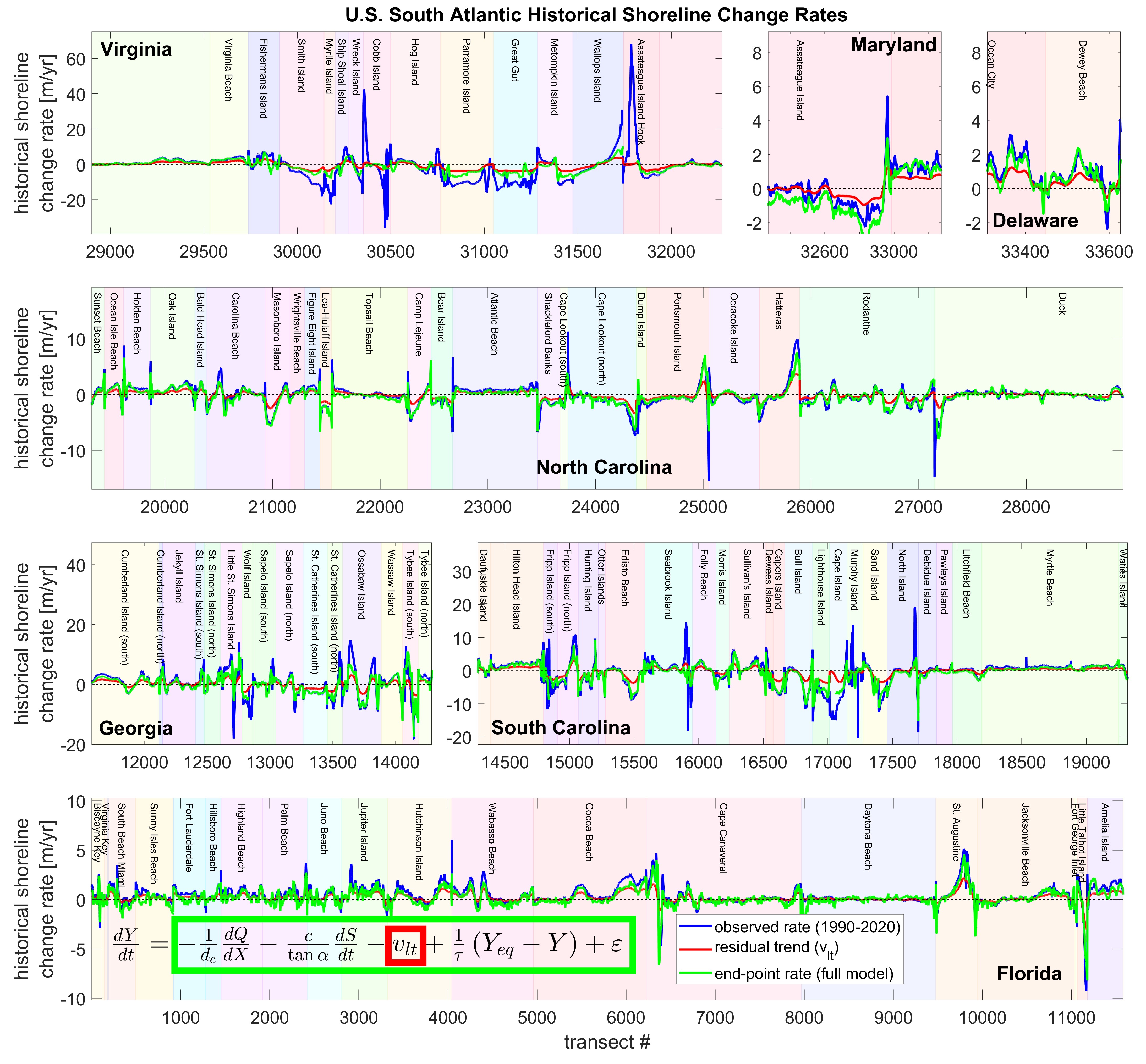


S7 - Comparison of the long-term, historical shoreline change rates ($\left(v_{\text{lt}}\right)_{0}$) derived from satellite shoreline observations from 1990 – 2020 (blue line), the modeled residual trend ($v_{\text{lt}}$; shown in red), and the end-point rate ($(Y_{2020}-Y_{1990})/30\text{ years}$) of the full model simulation (given Eq. 1 in Methods; shown in green) across the U.S. South Atlantic Coast (negative = erosion trend and positive = accretion trend). The x-axis on each of the subpanels represents the transect number, which increases sequentially from south to north along the coastline. Also, note that each of the subpanels is broken up by state and the y-axis limits vary between panels. The colored bands on each panel identify large littoral regions, which are generally enclosed by harbors, inlets, headlands, etc. The observed vs. modeled shoreline trends indicate that sections of coastline comprised of small barrier islands (e.g., Georgia, South Carolina, and northern Virginia) generally experience the most dynamic shoreline change and hence the largest historical trends, which are not always captured well in the model. On the other hand, sections of coastline that are relatively long, straight, urbanized (e.g., Florida, southern Virginia, Maryland, and Delaware), and occur in the middle of a littoral cell generally experience smaller historical trends and better comparisons between model (green) and observations (blue).


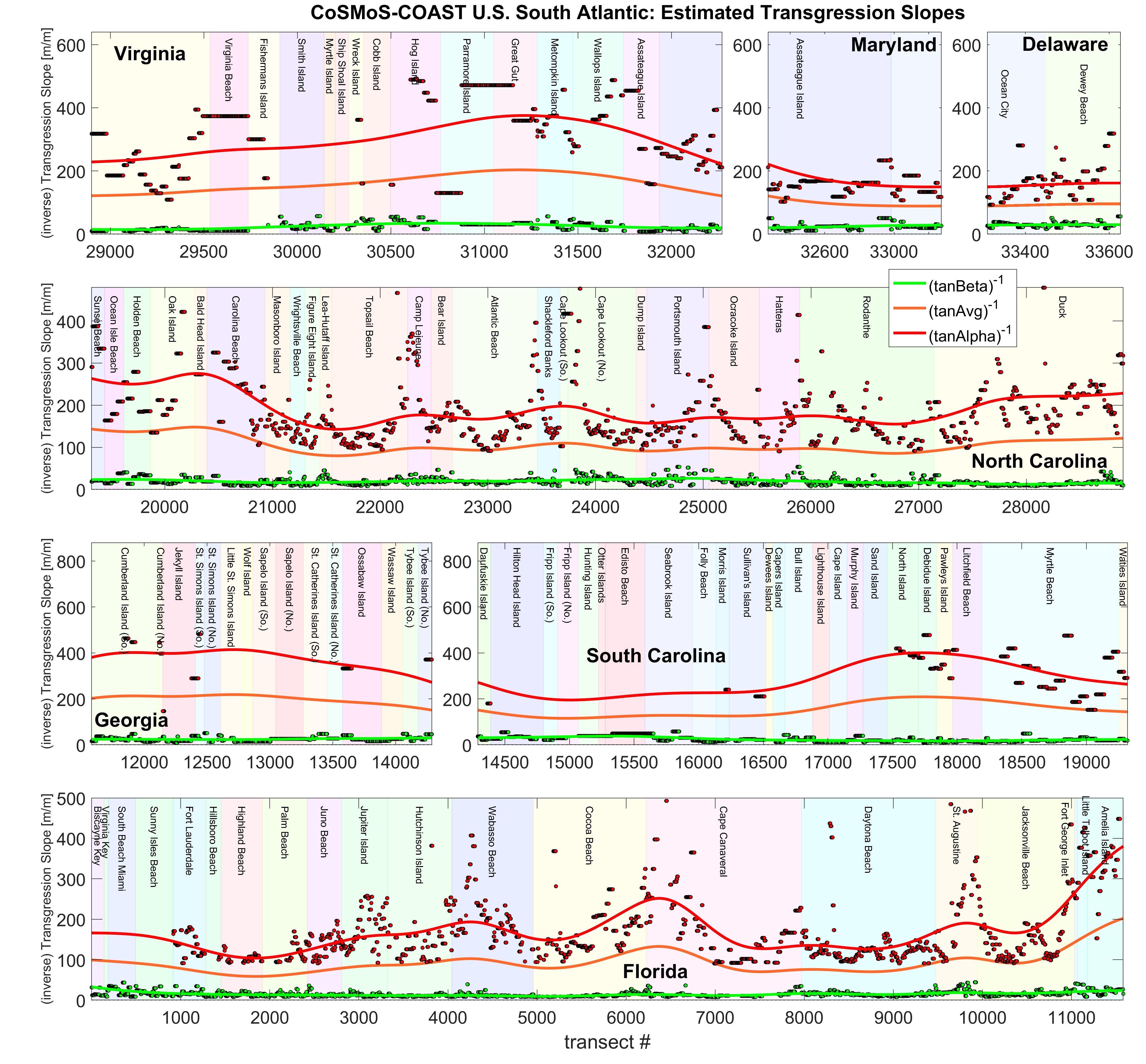


S8 - Transgression slope cases used in the current modeling application, derived from Mickey and Passeri (2022). The transgression slopes are derived from the slopes of beach elevation profiles (at a series of transects) that are extracted from 2-D digital elevation models (foreshore slope shown in green dots; active beach-profile slope shown in red dots). The current figure shows the (inverse of the) foreshore beach slope ($\tan\beta$ – in the green line; ‘steep’), the average slope of the active beach profile ($\tan\alpha$– in the red line; ‘gentle’), extending from the approximate depth of closure (~ 10 m) to the landward edge of the dune toe, and the average of the two (denoted as “tan Avg” – in orange; ‘intermediate’). The raw, observed beach profile slopes (dots) are regularized in the alongshore direction using a spline-based smoothing algorithm (Garcia 2010) for use in the model (solid lines). Note that each panel represents a different state on the U.S. South Atlantic Coast, and each panel can have different y-axis limits. Also note that while the foreshore beach slopes (with an average of ~1/20) are remarkably consistent in the alonghore direction, the average slope of the active beach profile (i.e.,$\tan\alpha$), is highly variable in the alongshore direction between about 1/100 and 1/400, with the most gentle transgression slopes generally occuring in regions with small barrier islands (e.g., Georgia, South Carolina, and northern Virginia).


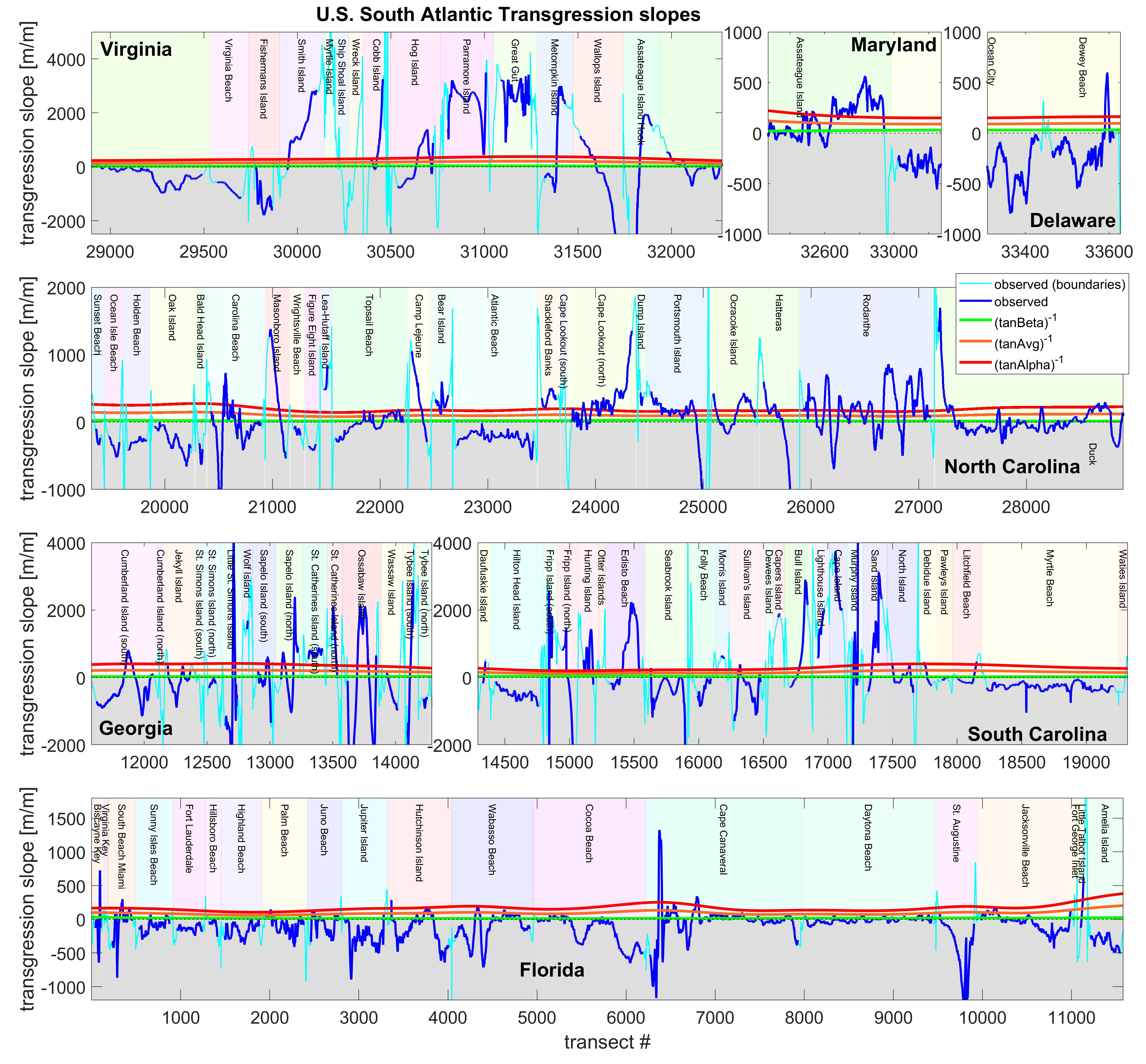


S9 – Comparison of the (inverse of the) observed transgression slope (dark blue lines) versus the transgression slopes derived from nearshore elevation-profile data used in the current modeling application (S8; ‘gentle’: $\left(\tan\alpha\right)^{-1}$– in red; ‘steep’: $\left(\tan\beta\right)^{-1}$ – in green; ‘intermediate’: “(tan Avg)^-1^” – in orange). Note that each panel represents a different state on the U.S. South Atlantic Coast, and each panel can have different y-axis limits. The observed transgression slopes at each transect are estimated from the ratio of the observed shoreline-change trend (S7) to the long-term rate of sea-level rise (i.e., 4 mm/yr, which is derived from the average of all tide-gauge-derived rates across the region). Applying this ratio to observationally estimate the transgression slope, as is often done in the literature, assumes that the historical shoreline trend is *only* caused by sea-level rise, which is generally not the case. Instead, the historical shoreline trend is likely caused by many coastal change processes (e.g., wave-driven longshore and cross-shore, nourishments, etc.). However, over a large coastal region, over a long period of time and with a reasonably high rate of sea-level rise, the commonly used methods to observational estimate shoreline transgression, analyzed here, may be considered sufficiently ‘optimal.’ Transects that are close to littoral cell boundaries (e.g., inlets/jetties) are shown in light blue colors since they will likely be disproportionally influenced by shoreline change factors other than sea-level rise (e.g., interruptions in longshore transport), and thus may be given less credence. Overall, the results, shown here, exhibit pronounced longshore variabiltiy in transgression slope, emphasizing that factors other than sea-level-driven recesion (which are, in theory, more alongshore uniform), are controling the variability in historical shoreline trends and thus observationally estimated transgression slopes. The high prevalence of negative transgression slopes (shown in gray boxes and associated with accretion trends), which are inconsistent with the occurrence of local sea-level rise, further suggests that shoreline-observation-based estimates of transgression slope are potentially fraught with complications arising from local processes/variability and beach nourishments. Only a very small portion of the U.S. South Atlantic Coast, e.g., Bear Island, Shackleford Banks, Cape Lookout, Portsmouth Island, Ocracoke, and Rodanthe (to a lesser degree), which are largely uninhabited barrier islands, seem to exhibit shoreline transgression rates that are comparable to the theory (i.e., the Bruun rule). We reiterate that the analysis shown in this figure does not indicate that sea-level driven recession is absent on the U.S. South Atlantic Coast. Instead, the results, presented here, likely reveal the difficulty of observationally estimating shoreline recession due to relatively small amount of historical sea-level rise from shoreline data across the region.


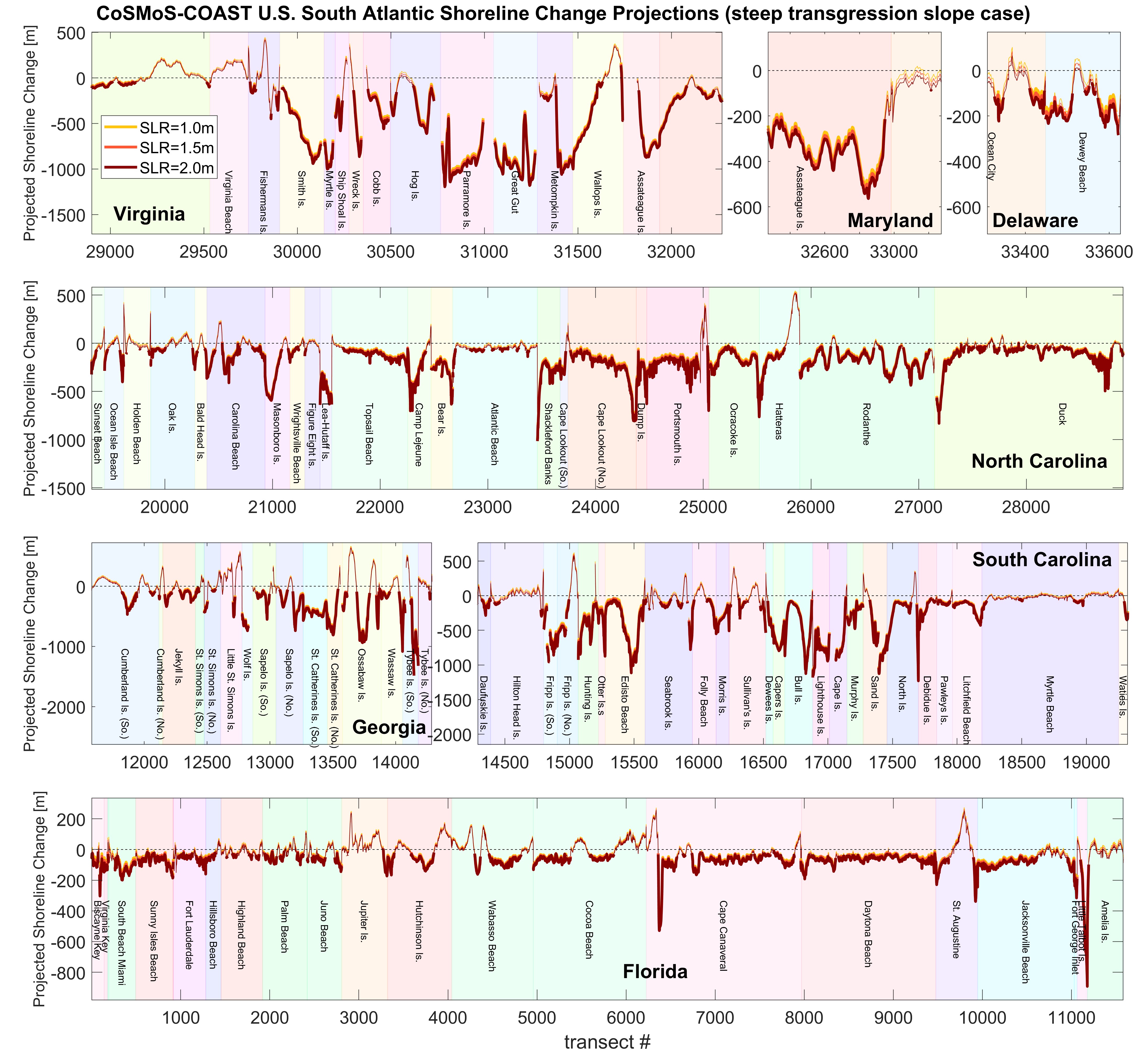


S10 - Model-projected shoreline positions in 2100 relative to the initial shoreline position (obtained from satellite-derived shoreline observations ca. 1990) versus transect number (which is numbered consecutively from south to north) for the *steep transgression slope scenario* (i.e., the green line in S8), which seeks to represent passive flooding of the beach profile without drastic landward shifts in the beach elevation profile. Note that each panel represents a different state on the U.S. South Atlantic Coast, which has different limits on the y-axis. The figure illustrates the shoreline-change simulations due to sea-level rise (SLR) projections of 1.0, 1.5, and 2.0 meters by 2100 in yellow, orange, and red colors, respectively, under the “no hold the line” and “continued accretion” management scenarios. Sections of the yellow, orange, and red lines in this figure are plotted as either thin or thick lines, which indicate that the projected shoreline position is either seaward or landward of the existing end of the sandy beach, respectively. Hence, the thin line segments indicate portions of the coastline where sandy beaches are still present in 2100, whereas thick line segments indicate where sandy beaches have become lost in 2100, while assuming a static back beach line. Note that in this figure, the differences in projected shoreline position between the 1.5 and 2.0 m sea-level rise scenarios are, on average, 10 and 20 m landward of the 1.0 m scenario, respectively, due to the 1/20 average transgression slope, used here. Hence, there is relatively little difference among the three sea-level rise scenarios (e.g., the yellow, orange, and red lines) visible on this scale, compared to the intermediate and gentle transgression slope scenarios shown in Figure 4 and S11, respectively.


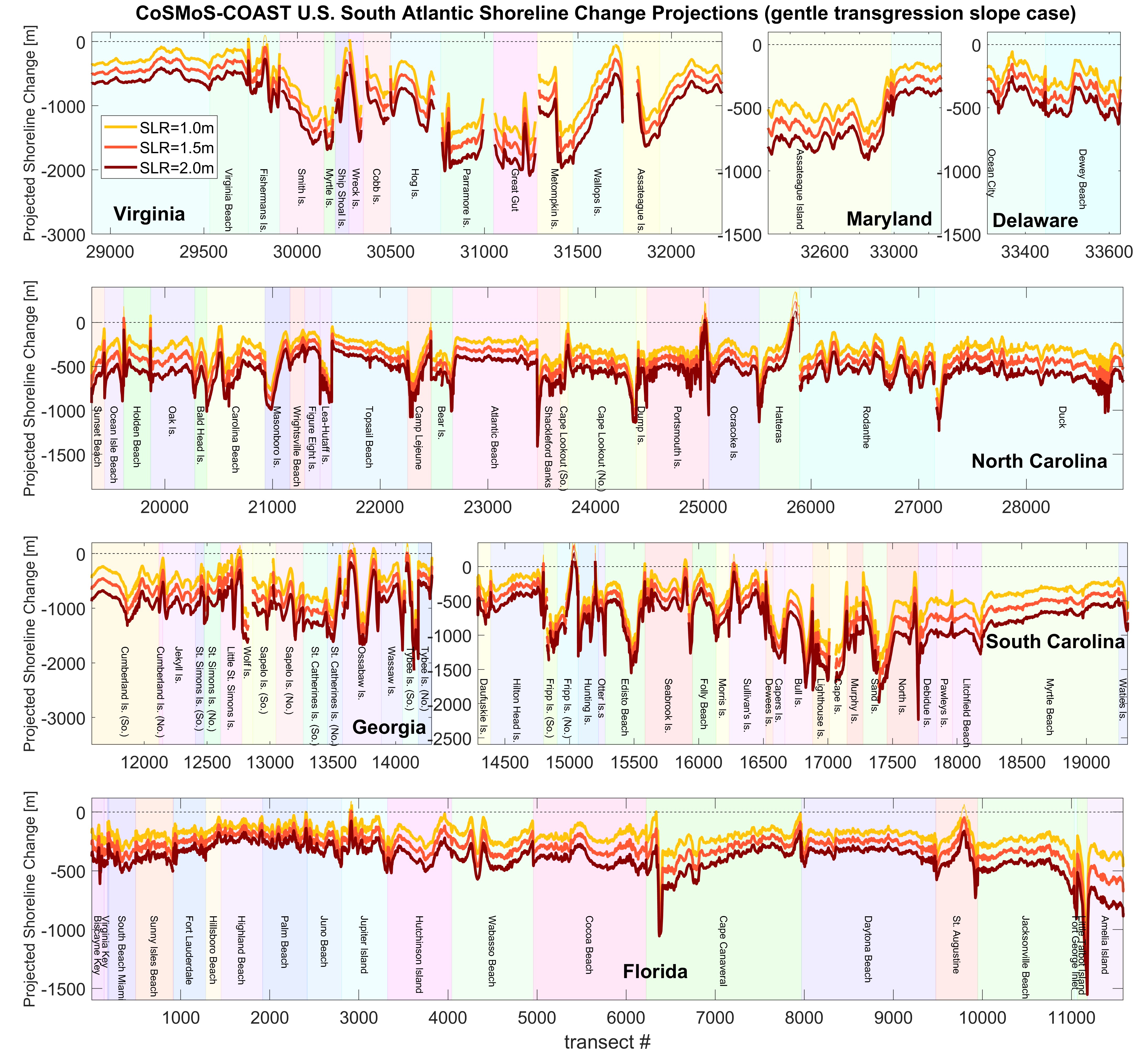


S11 - Model-projected shoreline positions in 2100 relative to the initial shoreline position (obtained from satellite-derived shoreline observations ca. 1990) versus transect number (numbered consecutively from south to north) for the *gentle transgression slope scenario* (i.e., the red line in S8), which represents the average slope of the active beach profile from the offshore depth of closure (~10 m) to the landward edge of coastal dunes. Note that each panel represents a different state on the U.S. South Atlantic Coast, which has different limits on the y-axis. The figure illustrates the shoreline-change simulations due to sea-level rise (SLR) projections of 1.0, 1.5, and 2.0 meters by 2100 in yellow, orange, and red colors, respectively, under the “unimpeded” and “continued accretion” model case. Sections of the yellow, orange, and red lines in this figure are plotted as either thin or thick lines, which indicate that the projected shoreline position is either seaward or landward of the existing end of the sandy beach, respectively. Hence, the thin line segments indicate portions of the coastline where sandy beaches are still present in 2100, whereas thick line segments indicate where sandy beaches have become lost in 2100, while assuming a static back beach line. Note that in this figure, the differences in projected shoreline position between the 1.5 and 2.0 m sea-level rise scenarios are, on average, 110.5 and 221 m landward of the 1.0 m scenario, respectively, due to the 1/221 average transgression slope, used here. Hence, there are relatively large differences among the three sea-level rise scenarios (e.g., the yellow, orange, and red lines).

**References**

1. Dietrich, J. C., Tanaka, S., Westerink, J. J., Dawson, C. N., Luettich, R. A., Zijlema, M., ... & Westerink, H. J. (2012). Performance of the unstructured-mesh, SWAN+ ADCIRC model in computing hurricane waves and surge. *Journal of Scientific Computing*, 52, 468-497.
2. Garcia, D. (2010). Robust smoothing of gridded data in one and higher dimensions with missing values. *Computational statistics & data analysis, 54(4), 1167-1178.*
3. Mickey, Rangley C., and Davina L. Passeri. (2022). "A Database of Topo-Bathy Cross-Shore Profiles and Characteristics for U.S. Atlantic and Gulf of Mexico Sandy Coastlines" Data 7, no. 7: 92. https://doi.org/10.3390/data7070092
4. Moin, P. (2010). Fundamentals of engineering numerical analysis. Cambridge University Press.
5. Roelvink, Dano. (2011). A guide to modeling coastal morphology. Advances in Coastal and Ocean Engineering: Volume 12. World Scientific.
6. Sweet, W.V., Hamlington, B.D., Kopp, R.E., Weaver, C.P., Barnard, P.L., Bekaert, D., Brooks, W., Craghan, M., Dusek, G., Frederikse, T., Garner, G., Genz, A.S., Krasting, J.P., Larour, E., Marcy, D., Marra, J.J., Obeysekera, J., Osler, M., Pendleton, M., Roman, D., Schmied, L., Veatch, W., White, K.D., and Zuzak, C., 2022. Global and regional sea level rise scenarios for the United States: updated mean projections and extreme water level probabilities along U.S. coastlines. NOAA Technical Report NOS 01. National Oceanic and Atmospheric Administration, National Ocean Service, Silver Spring, MD, 111 pp., <https://oceanservice.noaa.gov/hazards/sealevelrise/noaa-nos-techrpt01-global-regional-SLR-scenarios-US.pdf>
7. Vitousek, S., Cagigal, L., Montaño, J., Rueda, A., Mendez, F., Coco, G., & Barnard, P. L. (2021). The application of ensemble wave forcing to quantify uncertainty of shoreline change predictions. *Journal of Geophysical Research: Earth Surface*, 126(7), e2019JF005506.
